# Gimme that old time religion: the influence of the healthcare belief system of chiropractic’s early leaders on the development of x-ray imaging in the profession

**DOI:** 10.1186/s12998-014-0036-5

**Published:** 2014-10-28

**Authors:** Kenneth John Young

**Affiliations:** School of Arts; Senior Lecturer, School of Health Professions, Murdoch University, South Street, Murdoch, 6150 Australia

**Keywords:** Chiropractic, X-ray, Subluxation, History, Evidence-based practice, Religion

## Abstract

**Background:**

Chiropractic technique systems have been historically documented to advocate overutilization of radiography. Various rationales for this have been explored in the literature. However, little consideration has been given to the possibility that the healthcare belief system of prominent early chiropractors may have influenced the use of the diagnostic modality through the years. The original rationale was the visualisation of chiropractic subluxations, defined as bones slightly out of place, pressing on nerves, and ultimately causing disease. This paradigm of radiography has survived in parts of the chiropractic profession, despite lacking evidence of clinical validity. The purpose of this paper is to compare the characteristics of the chiropractic technique systems that have utilised radiography for subluxation detection with the characteristics of religion, and to discover potential historical links that may have facilitated the development of those characteristics.

**Discussion:**

Twenty-three currently or previously existing technique systems requiring radiography for subluxation analysis were found using a search of the internet, books and consultation with experts. Evidence of religiosity from the early founders’ writings was compared with textbooks, published papers, and websites of subsequently developed systems. Six criteria denoting religious thinking were developed using definitions from various sources. They are: supernatural concepts, claims of supremacy, rules and rituals, sacred artefacts, sacred stories, and special language. All of these were found to a greater or lesser degree in the publicly available documents of all the subluxation-based chiropractic x-ray systems.

**Summary:**

The founders and early pioneers of chiropractic did not benefit from the current understanding of science and research, and therefore substituted deductive and inductive reasoning to arrive at conclusions about health and disease in the human body. Some of this thinking and rationalisation demonstrably followed a religion-like pattern, including BJ Palmer’s use of radiography. Although access to scientific methods and research education became much advanced and more accessible during the past few decades, the publicly available documents of technique systems that used radiography for chiropractic subluxation detection examined in this paper employed a historically derived paradigm for radiography that displayed characteristics in common with religion.

**Electronic supplementary material:**

The online version of this article (doi:10.1186/s12998-014-0036-5) contains supplementary material, which is available to authorized users.

## Background

In 1910, fifteen years after the invention of chiropractic as well as the discovery of the x-ray, BJ Palmer, son of the founder of the profession, began vigorously promoting radiography to chiropractors [1]. Although there was some initial backlash to this invasive technology in a profession that focussed on manual therapy, [2] eventually it was embraced. In fact, chiropractic treatment systems have historically been documented to overutilise radiography [3–5]. However, overuse of diagnostic imaging is not unique to that profession [6–9]. Ionising radiation, even at the low levels used for diagnostic purposes, must be used judiciously due to the known risk of harm [10–12]. The rationale chiropractors have used to justify radiography has included diagnostic uncertainty, contraindications to manipulation/chiropractic adjusting, financial gain, discovery of occult congenital anomalies, routine screening, and biomechanical considerations [5,13–19]. The last item includes the discovery and/or quantification of tiny misalignments of spinal bones thought to impede the flow of vital energy through nerves, and known as chiropractic subluxations. Little evidence exists in the peer-reviewed literature to support this last use of ionising radiation [20,21]. Therefore, techniques using it for this purpose would seem to be driven by something other than purely rational, evidence-based clinical reasoning. Belief in the absence of evidence is essentially the operating system of religion. This observation raised the question as to whether the technique systems that employ plain radiographs for subluxation detection exhibited any similarities to religion.

There is a historical schism in chiropractic around the issue of radiography [22]. Some practitioners advocate a purely diagnostic approach, using radiography for the detection of pathology such as fracture or tumour, some advocate radiography purely for subluxation detection, and some for both. This debate was acknowledged as early as the 1930s. Waldo Poehner wrote about the advantages of using chiropractic radiographic views for chiropractic purposes and standard radiographic views for the detection of pathology, encouraging each camp to see the merit in each other’s arguments [23]. The paradigm that the primary use of radiography is to discover or quantify chiropractic subluxations has survived over a hundred years in parts of the chiropractic profession [1]. Although evidence-based guidelines regarding ionising radiation exist, [14,24–26] the profession does not universally endorse them [27,28].

The belief system around the larger issue of the chiropractic subluxation itself has been addressed in an attempt to help practitioners understand the basic concepts of science and to encourage a profession-wide move toward evidence-based care [29,30]. In addition, although the literature contains a number of papers on the subject of religion and dogma in chiropractic, [29,31–37] little consideration has been given to potential religious connotations specifically regarding the use of radiography for subluxation detection.

A short digression into the concept of the subluxation may be useful to readers not familiar with it. The vertebral subluxation is the traditional basis of the chiropractic profession. DD Palmer, who ‘discovered’ chiropractic in 1895, used the term to describe a ‘bone out of place’ that impinged the flow of vital energy, which he called ‘Innate’ or ‘Innate Intelligence’, to organs throughout the body, thereby causing dis-ease, and disease. This concept is a variant of the ‘one cause’ theories of healthcare that were common at that time [38]. The entire spectrum of ill health in mankind was attributed to altered transmission of nerve energy from slightly displaced bones affecting various nerves. According to BJ Palmer:

‘Chiropractic had long maintained… that a vertebral subluxation produced a pressure upon nerves which interfered with the normal and free transmission of mental impulses between the brain and its body; that this unequal state of balance between generation, transmission and expression produced disease. That the *sunnum bonum* of all life and death, health or disease issues pivoted around a study of the correct or incorrect position of vertebrae’ [39].

The idea that maximum expression of life can be obtained only when the bones of the spine (or a portion of the spine) are in the ‘correct’ place so that life force may flow unimpeded is a form of vitalism. In 1993, Gaucher-Peslherbe described the subluxation concept advanced by DD Palmer as being based on a moral conviction rather than a scientifically testable theory [40]. The credulity shown by chiropractors towards vitalistic ideas was noted by Keating who wrote that most members of the profession, at least in the 1920s, were ill-equipped to differentiate various epistemologies, such as uncritical rationalism versus casual empiricism versus the scientific method [2]. This propensity may not be limited to the early 20th century. Moore described it more comprehensively and charitably: ‘Contemporary chiropractic belief systems embrace a blend of experience, conviction, critical thinking, open-mindedness, and appreciation of the natural order of things’ [38].

The purpose of this paper is to compare the characteristics of the chiropractic technique systems that have utilised radiography for subluxation detection with the characteristics of religion, and to discover potential historical links that may have facilitated the development of those characteristics.

## Methods

A six-part strategy was designed. First, the writings of early chiropractic leaders, including DD Palmer and BJ Palmer were reviewed for their religious content. This was seen as relevant, because it was as indicative of the intellectual environment in which they operated. As the self-titled ‘Discoverer’ and ‘Developer’ of chiropractic, respectively, they and their inner circles were influential in the profession [41,42]. Their modes of thinking necessarily affected that of their followers. Second, since BJ Palmer was instrumental in introducing radiography to chiropractic, his paradigm for radiography was used as a starting point. Evidence was gathered from his writings on the topic, specifically, *The Subluxation Specific The Adjustment Specific,* [43] *Answers*, [44] *Chiropractic Clinical Controlled Research,* [45] *The Science of Chiropractic,* [46] and Palmer’s Introduction to EA Thompson’s *Chiropractic Spinography,* [1] as well as quotes from historically scholarly secondary sources such as Keating’s *BJ of Davenport*, [2] Dye’s *The Evolution of Chiropractic*, [47] Wardwell’s *Chiropractic: History and Evolution of a New Profession*, [48] and Moore’s *Chiropractic in America* [38]. Third, a list was created of techniques that used plain radiography for the purpose of finding chiropractic subluxations. This was done by searching the internet, entering into Google the key phrases ‘chiropractic x-ray’, ‘chiropractic radiography’, ‘chiropractic x-ray line drawing’, and ‘chiropractic subluxation’. PubMed was searched with the term ‘chiropractic and technique and x-ray’. Cooperstein and Gleberzon’s *Technique Systems in Chiropractic* [49] was used as a source. In addition, two experts in the fields of chiropractic history and technique systems were consulted. In order to be included in this study, current or previously existing chiropractic technique systems had to be named systems. They also had to have specific diagnostic/analytic protocols that included the visualization of subluxations on plain radiographs. The practices of individual chiropractors or groups that used modifications of the techniques were excluded. Fourth, a definition of religion was developed from works on comparative religion and anthropology. This was then modified to be practically applicable with clearly defined, specific parameters. Fifth, the publicly available documents of the technique systems, including their textbooks, published papers, magazine articles and official websites were examined for evidence of the religious characteristics developed as described above. Finally, the information was collated and examined. A list of the six characteristics was created, and evidence from each technique system entered into it; the list was then divided into the six appendices of this paper. Two tables were developed. One is a list of all the technique systems (see Additional file 1: TableS1), and the second is a chart with six headings, each corresponding to a religious characteristic, and a brief synopsis of the evidence from each technique system was entered under each heading, the technique systems appearing in alphabetical order under each heading (see Additional file 2: Table S2). The characteristics of the radiographic paradigm of chiropractic’s early leaders were then compared to that of the subsequently developed technique systems, and historical links demonstrated.

## Discussion

### Religiosity in early chiropractic leaders

The aetiology for the environment within chiropractic that fostered belief over evidence can be demonstrated in the books and papers left by the founders. DD Palmer wrote about his religious convictions in papers published after his death: ‘I believe, in fact know, that the universe consists of Intelligence and Matter. This intelligence is known to the Christian world as God’ [50]. Palmer continued with his explanation of the way Intelligence or spirit is expressed through the nervous system, and that all physical and mental functions are modified by the physical condition of the body. He concluded with this: ‘a correct understanding of these principles and the practice of them constitute the religion of chiropractic’ [50]. DD Palmer felt that he had a sacred duty in relieving people’s subluxations: ‘Knowing that our physical health and the intellectual progress of Innate depend upon the proper alignment of the skeletal frame, we feel it our bounded duty to replace any displaced bones so that physical and spiritual health, happiness, and the full fruition of earthly life may be fully enjoyed’ [51]. DD Palmer even explicitly considered turning chiropractic into a religion, as Mary Baker Eddy had done with Christian Science [52].

**Figure 1:**
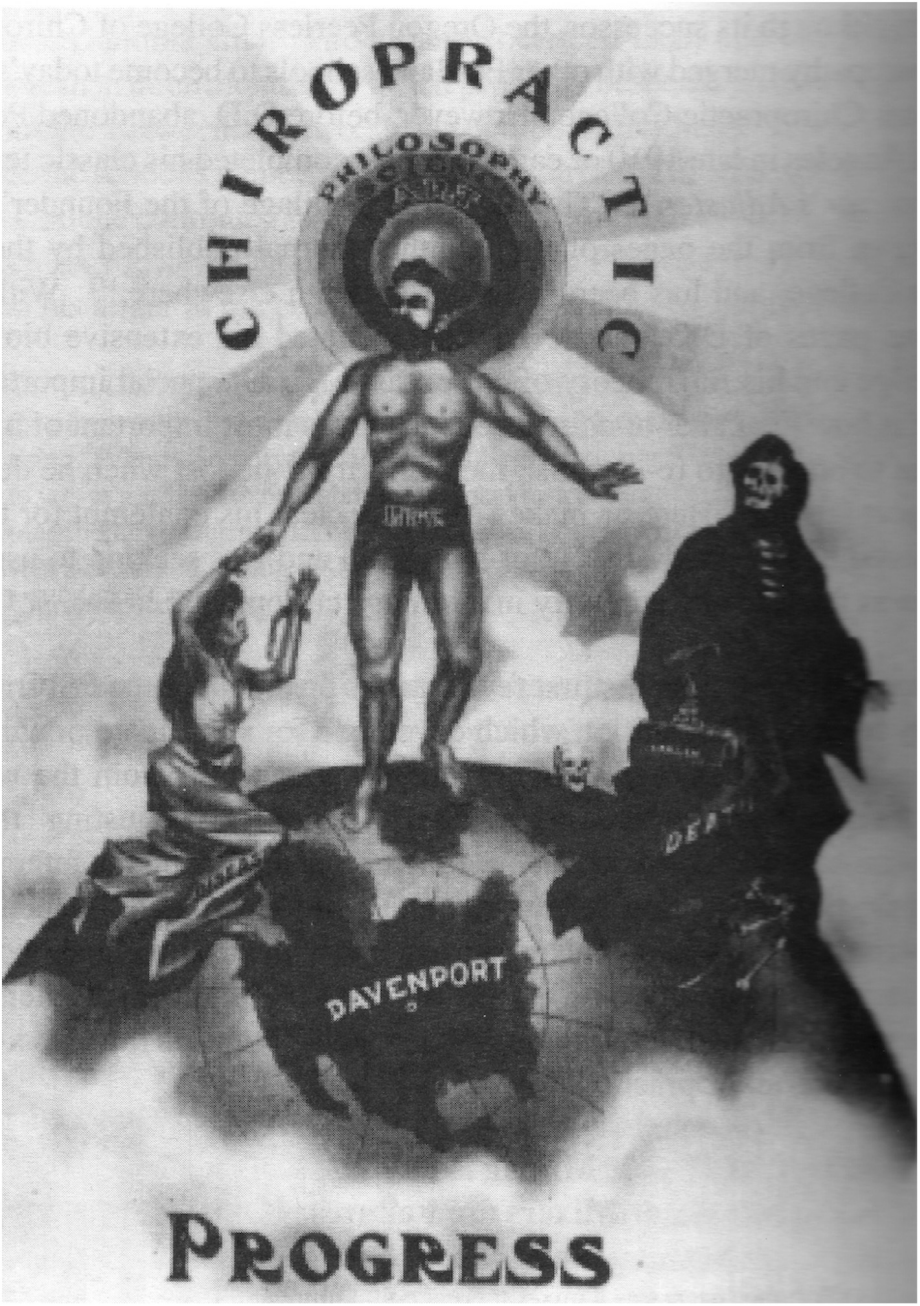
**BJ Palmer as healer of the sick, fending off Death, with halo-like emanations from his head, 1910 (with permission from Special Collections and Archives, Palmer College of Chiropractic).**

After his father’s death, BJ Palmer expanded on the religious theme. In 1915, feeling the effects of medical attacks on the profession, he compared himself to Jesus Christ. He wrote a detailed description of the crucifixion scene, finishing with: ‘Father, forgive, they know not what they do. It behoves us all to be forgiving. I have tried to be charitable to those who would do otherwise’ [2]. He continued this theme over time. John J Nugent’s advocacy of improved academic standards earned him expulsion from Palmer’s school in June 1922. BJ accused him of disloyalty, disrespect and insult to the President as well as making statements derogatory to the institution [53]. Eventually Nugent was influential in the development of national standards for chiropractic education, and Palmer took to calling him the anti-Christ of chiropractic, [53] which seemed to imply Palmer as the Christ. BJ was even known to repeatedly state: ‘The first man that cured by laying on of hands they crucified’ [48]. Keating noted religious overtones from the early days of chiropractic, [2] citing an illustration in the magazine *The Chiropractor* in 1910 that depicted BJ Palmer nearly nude and improbably muscular, with a halo emanating light rays, fending off death and giving aid to the ill (Figure 1).

Another overtone of religion appeared on the masthead of Palmer’s *Fountainhead News,* a weekly newsletter written ‘by BJ himself’. Volume 5 number 19, issued on 24 June 1916 reworked the Gregorian calendar, dating the world (of chiropractic, at least) from 18 September 1895, the supposed date of the first adjustment. The notation for 1916 became AC 21, with AC standing for ‘After Chiropractic’, a parallel to the Christians’ AD, *Anno Domini* [2].

Some of Palmer’s ‘disciples’ held strong convictions, conveying the chiropractic belief with great fervour. In 1927 a Palmer alumnus and teacher in chiropractic philosophy at his alma mater, produced the *Chiropractic Textbook* [54]. In it, author Ralph W Stephenson elucidated his thirty-three principles of chiropractic. He declared explicitly that chiropractic could not be tested by science, but rather came from God and therefore could not be refuted. ‘By assuming a major premise, that there is a Universal Intelligence which governs all matter, every inference drawn from that major premise and subjected to specific scrutiny, stands the test’ [54]. BJ Palmer was effusive in his praise for this work, writing in its introduction ‘Of ALL the books written and compiled on Chiropractic Philosophy, this is by far the best, not excepting my own’ [54].

Historian J Stuart Moore also noted a religious thread running through some of the early practitioners of chiropractic. For them, BJ, who was happy to take the role of messiah, provided a focus for an undefined or unfulfilled desire for answers to the mysteries of life and health:

‘Chiropractic drew believers with similar yearnings but with at least two clearly distinguishable temperaments that determined how their allegiance to chiropractic would develop. The first type came with deep spiritual longings but no strongly fixed religious faith… chiropractic became a substitute religion, supplying a comprehensive explanation of reality, complete in itself with claims of divine powers, suffering saints and martyrs, and sacred writings and utterances from prophets (the Palmers) who provided the ultimate source of authority – all wrapped in a millennial eschatology’ [38].

Another group, Moore noted, were conventional Christians, who made chiropractic fit in with their pre-existing beliefs. He indicated the ease with which chiropractic believers could substitute Palmer’s Universal Intelligence as the Christian God and Innate Intelligence as the human manifestation of the divine [38]. Improvements in health due to chiropractic ministrations meant that the chiropractor was, quite literally, the hand of God, revitalising the sick with heaven-sent energy.

BJ began holding an annual homecoming, which he called a lyceum, at the Palmer School starting in the year 1914 [2]. These were part seminar, part spectacle, with celebrities of the day attending as guests and other entertainment. Keating characterised them as the ‘traditional opportunity to bedazzle and inspire the faithful to the cause’ [2]. Like an old-time tent revival, these gatherings helped renew faith in chiropractic and in Palmer as the leader [2].

These are just a few examples, cited to demonstrate the environment of belief, rather than scepticism, that was disseminated from the highest levels of the early profession. It is reasonable to argue that this situation at least allowed, and even encouraged some subsequent technique originators to accept the basic premises of chiropractic without question.

### BJ Palmer and the historical chiropractic paradigm for radiography

**Figure 2:**
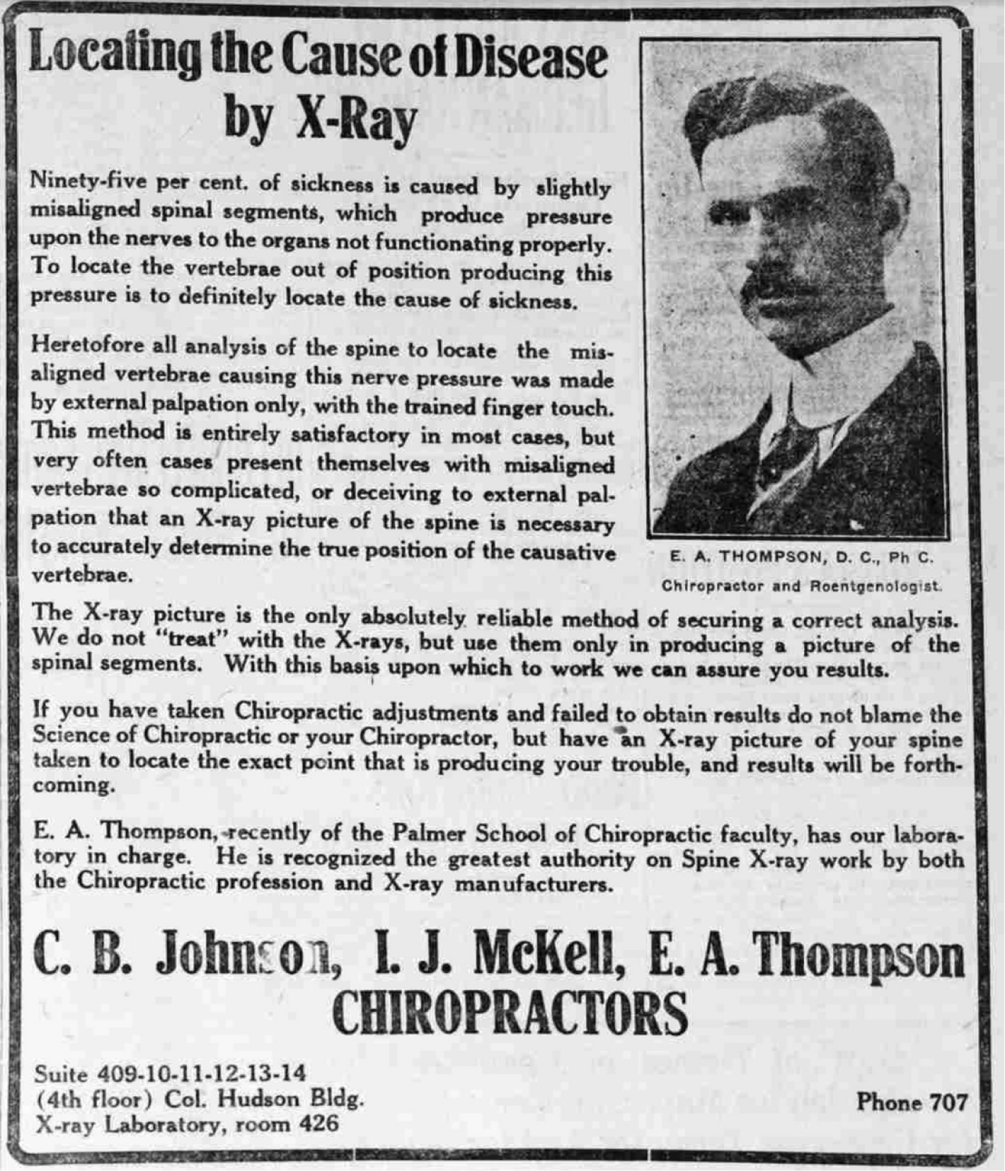
**Advertisement in the Ogden (Utah) Standard, 1917.**

BJ Palmer bought the first x-ray machine for the Palmer School of Chiropractic in 1910, theorising that since this new technology allowed the visualisation of bones within the body, subluxations could now be ‘proved’. This would legitimise the core theory of chiropractic. ‘The advent of the X-Ray [sic] into Chiropractic was to prove that vertebral subluxations did actually exist and could be so proven with the aid of the X-Ray make this visible to the eye’ [1]. Palmer then advanced the technology as the critical component of chiropractic practice: ‘The spinograph means the difference between failure and success: No results and results. Guess and knowledge. Doubt and positiveness. Theory and fact’ [1]. Essentially, BJ Palmer used x-ray to help convince people of the veracity of his claims about subluxation. There is evidence that was effective at this task, because some chiropractors began espousing BJ’s rhetoric. For instance, a 1917 Utah newspaper advertisement by chiropractors CB Johnson, IJ McKell and Palmer’s former spinographer, EA Thompson, began with the statement that ‘95% of sickness is caused by slightly misaligned spinal segments’ and that ‘the X-ray picture is the only absolutely reliable method of securing a correct analysis’ [55] (Figure 2). The more recently developed system Chiropractic BioPhysics utilised a credulous approach in their research, gathering evidence for spinal subluxation as visualised on radiographs, an assumption granted prior to commencement of data collection: ‘The **PCCRP** [Practicing Chiropractors’ Committee on Radiology Protocols] guidelines are the Evidence Based Support for subluxation analysis via x-ray as performed by a large percentage of practicing chiropractors’ [27]. Others claimed to refine methods without ever questioning the basic premise of the primary role of the subluxation in the creation of ill health; much in the way theological studies may undertake investigation of a deity without ever questioning its existence. For example, Zimmerman proclaimed that due to his refinements in radiography and treatment methods, ‘[the chiropractor] is trained to place the tip of the machine so accurately as to do that which has never been done before’ [56]. ‘The Gonstead Chiropractor goes beyond what many chiropractors consider a spinal assessment by conducting a thorough analysis of your spine using five criteria to detect the presence of the vertebral subluxation complex’ [57]. It seemed that in contrast to evidence-based healthcare, the epistemology of ‘appeal to authority’ was generally found to be highly valued in these systems.

The assertion that vertebral subluxation is the primary cause of ill health is a dogmatic, religious-like position, rather than the basis for a system that responds to changes in evidence in order to provide the best care for a variety of conditions. This belief system extends beyond the use of radiography to the idea of the subluxation in general. However the focus of this paper will be on the elements of the belief as reinforced by radiography. This group by no means represents all of chiropractic, and it should be noted that the American Chiropractic College of Radiology does not endorse this use of ionising radiation, [22] nor do the mainstream radiology guidelines to which many chiropractors adhere [26].

### The techniques that advocate radiography for subluxation analysis

Twenty-three specific, named technique systems were found (see Additional file 1: TableS1), comprised of: Advanced Orthogonal, Applied Spinal Biomechanical Engineering (ASBE), Applied Upper Cervical Biomechanics (AUCB), Atlas Orthogonality, Blair, Chiropractic BioPhysics (CBP), Cowin Upper Cervical Orthogonal, Duff, Gonstead, Grostic, Kale, Knee-Chest Upper Cervical Specific, Logan Basic, Mears, National Upper Cervical Chiropractic Association (NUCCA), Orthospinology, Palmer Upper Cervical Specific (also known as HIO, Hole-in-One or Toggle Recoil), Pettibon, Spinal Orthopedic Neurological Advancement and Research (SONAR), Spinal Stressology, Sutter and Zimmerman. The Palmer original full spine technique was not found to exist as a specific technique today. Pierce-Stillwagon deserves a specific note. Created in 1975, it only briefly required imaging, and then reduced the emphasis on radiography, preferring thermography to locate subluxations [49]. Walter Vernon Pierce subsequently split from Glenn Stillwagon [49] and not enough information on Stillwagon technique was found to meet the inclusion criteria, but the Pierce Results system was found, and will be included below. One paper [58] listed two additional names as techniques, Hildebrandt and Wernsing, but not enough information about their methods or history was found to meet the inclusion criteria. In addition, AA Wernsing’s ideas seem largely to have been incorporated into BJ Palmer’s Upper Cervical Specific technique [43].

### Characteristics of religion

There are a great many definitions of religion, most of which focus solely or primarily on the idea of belief in a deity or guiding hand in life, [59–62] but sociologically based ones exist [63]. As has been noted elsewhere, [64] the variety of perspectives on the topic suggests that religion is difficult to define. Specific characteristics were sought for this paper, in order to have an operational definition. This would allow comparison of the characteristics to the concepts and devices found in the technique systems. Albanese [64] addressed chiropractic specifically, and defined religion as a ‘way of organizing reality and relating to it’ [65]. When referring to a system of natural healing, that is, non-pharmaceutical and non-surgical, she employed the term ‘nature religion’ [65]. As will be seen, the chiropractic technique systems examined in this paper did seem to organise reality according to their own standards, eschewing mainstream principles of healthcare, and adopting their own versions of a uniquely chiropractic paradigm for radiography. For the purpose of this paper, an essentially behavioural definition was created, drawing partly from the above references and modified by the author’s observations of prominent religions. The definition consisted of six characteristics: 1) supernatural concepts relating to a belief in a supreme power, 2) claims of supremacy to others that are similar, 3) rules that must be followed, including carrying out rituals, 4) sacred artefacts, often utilised in the rituals, 5) sacred stories, and 6) special language. Evidence for each of these characteristics as observed in religions is given as they are individually discussed below. The religious characteristics of these techniques ranged from striking to mild but were not absent from any of the systems.

Evidence-based chiropractic attempts to use diagnostic imaging as well as vocabulary and concepts of health and disease conventional with the medical establishment. However, the above-listed elements of religion were demonstrably present in the publicly available documents of chiropractic technique systems that asserted vertebral subluxation as the primary or sole cause of disease, and that utilised radiographic analysis as their main diagnostic test for this lesion.

### Characteristics of religion in the technique systems

#### Supernatural concepts relating to a belief in a supreme power

Supernatural concepts in religions are usually thought of as the idea of a superior being with some degree of influence on earthly life, or a spirit or life force, something that transcends the laws of nature. For this paper, chiropractic supernatural concepts were vitalism and an ethereal feeling of being ‘called’ to the profession. Vitalism is the idea of vital energy (life force), called Innate Intelligence or just Innate by the Palmers, the key to health that may be blocked by small misalignments of bones near nerves. Therefore, references to ‘nerve interference’ preventing full expression of human health were considered vitalism, and therefore supernatural. Some techniques directly referenced BJ Palmer, such as Grostic, where ‘misaligned vertebra(e) impinge the flow of innate intelligence and cause illness, or what BJ Palmer later referred to as dis-ease’ [66]. Another example was Applied Upper Cervical Biomechanics: ‘A chiropractic doctor detects and corrects these spinal misalignments (vertebral subluxation causing nerve interference), restoring normal nerve impulses to the body allowing the body's own innate healing capabilities to function optimally’ [67]. Some referenced Palmer’s concepts more obliquely, without using the terms ‘Innate’ or ‘Innate Intelligence’ but expressing Palmer’s ideas nonetheless. For example, Cowin: ‘Our procedures are based on the traditional chiropractic concept of vertebral subluxation’ [68]. Or Blair: ‘The nervous system controls and regulates all parts and functions in the body. When there is nerve interference (subluxation) your body loses the ability to properly self regulate and heal itself. The result can be pain and illness. Blair upper cervical care is often the key to people regaining and maintaining good health’ [69]. Some appeared to be peripheral on the topic, but nevertheless expressed the same idea, like Chiropractic BioPhysics, who claimed that their mission was to ‘correct human spines and alleviate human suffering’ [70].

A calling to the profession was expressed in various ways. Techniques like Spinal Orthopedic Neurological Advancement and Research (SONAR) link the idea for the technique directly to a deity: ‘Through the grace of God, Dr. Elliott has developed the SONAR instrument’ [71]. Others expressed the idea that their practice was a form of worship, like Advanced Orthogonal: ‘we recognize that the human body is fearfully and wonderfully made, and our desire is to honor the Creator of this dynamic body through our approach to health care’ [72]. Still others viewed their duty within the profession to include a kind of evangelism: ‘Our Mission is to perpetuate the teachings of Dr. Clarence S. Gonstead, fund chiropractic research, and encourage cooperation and camaraderie amongst all who practice the Gonstead technique’ [73]. The linkage between profession and religion is expressed outright by a few: ‘The Kale Foreign Messenger has been in over 9 countries doing missionary work’ [74]. The full evidence from the writings of each technique is found in Appendix 1. All the techniques demonstrated some element of supernatural thinking.

In addition to text referring to the supernatural, a few techniques employed religious imagery in their official publications. Otherworldliness was implied by a picture on the Gonstead website, showing the originator of the technique with hands clasped, wearing a white shirt with high, priestly collar, staring beatifically into the distance, a halo emanating from his upper body [75]. NUCCA had a picture of a sunrise over a road heading off into a heavenly looking horizon [76]. Duff used a chiropractic angel, similar to a medical caduceus, with rays like sunshine emanating from it [77]. It should be noted that there is no evidence that the chiropractic angel was invented with a supernatural implication. Created for a public relations campaign by the American Society of Chiropractors around 1928, many different forms of this symbol have existed over the years, adopted and modified by chiropractic practices and institutions with widely varying approaches to the profession [78].

Other techniques used multimedia that evoked religious comparisons. Orthospinology’s website had a video entitled ‘Changing the World, one spine at a time’, which used music that sounded like a church choir singing over electronic dance music [79]. Whether implied or explicit, these techniques used words, images and sounds often more evocative of religion than mundane and earthly science.

#### Claims of supremacy

Many religions claim that theirs is the one true path to enlightenment, something Runzo [80] called religious exclusivism [80]. This characteristic was expressed in the writings of the technique systems using radiography for subluxation detection. These were all similar to the attitude BJ and DD Palmer had when they declared chiropractic to be the one cure for the one cause of disease. Assertions of superiority were found for all the techniques, and many claimed it by precision of radiographic analysis or adjustment. This set them apart not just from medical methods, but also from other chiropractic techniques whose methods were similar to their own. In terms of tiniest misalignments discovered, Advanced Orthogonal referred to: ‘Digital x-ray analysis to measure misalignments to the 1/100th of a degree’ [81]. Cowin averred that their methods would uncover displacements ‘as small as 0.75 degrees (or, translated into linear measurements, each as small as 28 thousandths of an inch or 0.7 mm)’ [82]. Others claimed superiority due to research, for instance ASBE asserted ‘12 volumes of A.S.B.E. research discoveries’ [83]. AUCB boasted ‘15 years of unprecedented research’ [84]. Many claimed simple, overall superiority: ‘the secret of health and longevity has been solved… in Logan Basic Technique more than by all other asserted advances in healing methods since the beginning of time’ [85]. SONAR claimed its system as ‘far superior to other upper cervical techniques’ [71]. Orthospinology declared itself the ‘greatest health care procedure in the world’ [86]. See Appendix 2 for examples from each technique.

#### Rules and rituals

**Figure 3:**
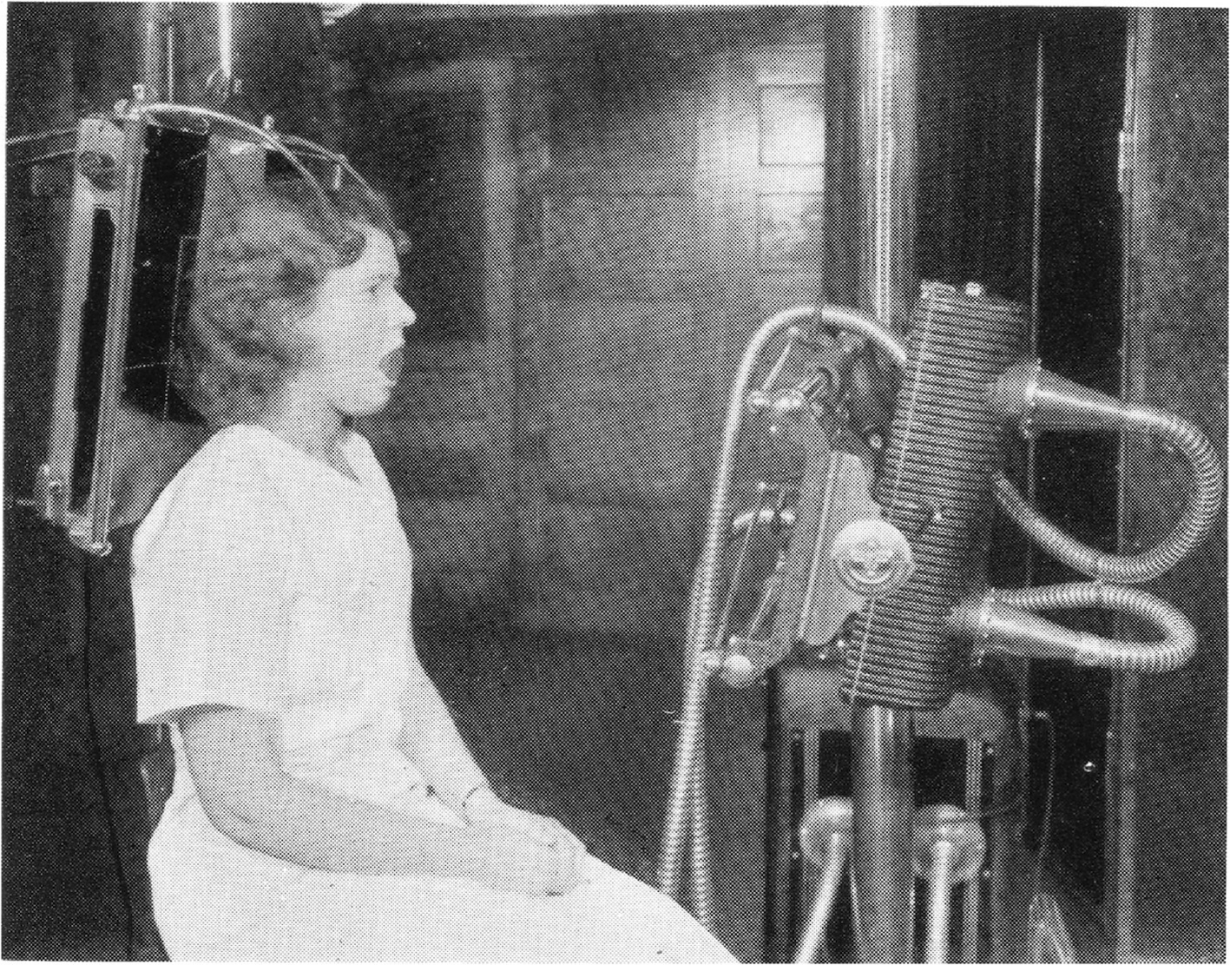
**Body positioning devices used in preparation of x-ray for subluxation analysis.** (Remier, PA, *Modern X-ray Practice and Chiropractic Spinography*. 1^st^ ed. Davenport, Iowa: Palmer School of Chiropractic, USA, 1938, 260).

Examples of rules followed in religions include such things as the Ten Commandments, Jewish Kosher food restrictions, and Islamic fasting during Ramadan. There are two rules that are common to all these chiropractic techniques: the chiropractic subluxation is the primary basis of disease in humans, and plain film radiographs must be obtained in order to detect or define it. The same set of images was claimed to be necessary, differing according to each technique’s rules, regardless of their symptoms. In the upper cervical systems, several views of the neck and lower part of the skull were taken, sometimes with elaborate patient positioning devices (Figure 3). For many of the systems, post treatment radiographs were also required to confirm replacement of the bones. In the full spine systems, radiographs of the entire spine, and often the pelvis were required to search for subluxations. In a best-case scenario for the patient, the images would be examined for pathology in addition to subluxation/postural change. However even this amounted to screening for disease with x-rays. This practice has not been supported in the healthcare literature for over half a century, due to the potential negative consequences of ionising radiation and the low probability of uncovering significant findings [87–93]. Therefore, any of the more recent techniques that advocated radiography for subluxation detection did not follow the evidence for best practice of radiography, but rather their own belief system of healthcare.

Turner [94] defined a religious ritual as ‘a stereotyped sequence of activities involving gestures, words, and objects, performed in a sequestered place, and designed to influence preternatural entities or forces on behalf of the actor’s goals and interests’ [94]. Ritual behaviour has been noted for its rigidity, repetition, and apparent lack of rational motivation [95]. The effect on those that partake of religious rituals has been described as bringing order, comfort, and organization through shared familiar symbols and patterns of behaviour [96]. Religious rituals include baptism, lighting candles at Hanukkah, reciting seven vows while taking seven steps at the Hindu wedding ceremony. The common ritual in all the technique systems was the taking of the radiographs; although the areas imaged and the analytical procedures vary to some degree, they all involved finding a slightly malpositioned vertebra or postural alteration. The images were often used to explain the issues of subluxation or abnormal posture as well as their dire consequences [97,98]. The rules and rituals of the systems indicated a rigidity of methods applied uniformly to all patients, rather than the customized exam and treatment that one would expect from other types of health care practitioners. This may have represented a form of indoctrination. Ritual gives artefacts value and defines the sacred within a community [99].

Many of the techniques also required post-treatment images as routine, with a stated goal of visualising the bone back in the correct place. Post-treatment radiography is a questionable practice, since it is of no benefit to the patient, but does give a dose of ionising radiation. However, this practice may be perceived as part of the ritual indoctrination of patients. The entire radiographic procedure could arguably perform the function of creating a stronger bond between practitioner and patient, as well as enhancing belief in the technique system for both. See Appendix 3 for evidence regarding x-ray rules and rituals in all the techniques.

#### Sacred artefacts

Sacred artefacts varied somewhat from technique to technique, but they all had in common the x-ray machine and the radiographs. These artefacts were used as powerful symbols, around which was woven the story of the technique. Comparison can be made to the crucifix is a symbol upon which the story of Christianity relies, or a turban as an object of strong religious significance for a Sikh. These artefacts help create or augment the ritualistic experience, and thereby indoctrinate new members and perpetuate the system of religion.

In the context of techniques that required radiography for subluxation detection, the x-ray machine allowed the direct visualisation of the health-depleting subluxation (or postural alteration), so its status was elevated to the highest (holy) level. Similarly, the radiographs themselves (whether plain film hard copy or digital images on a screen) demonstrated the visible manifestations of the lesion upon which these practitioners founded their diagnosis and treatment. Their service in reinforcing the reality of the technique systems was likely very strong. People will always be greatly stimulated by the visible. ‘Seeing is believing’ is a cliché because it is largely accurate. Therefore, to be able to point to a radiographic image and tell a person in pain that his/her problem is ‘right here’ must be a strong inducement to believing.

In some cases, special head clamps or other body positioning devices specific to the technique may have been used for the radiography. The more elaborate the ritual, the more persuasive it may be, [100] and this precept seemed to have been used by some of these systems. For example, ‘Pettibon x-ray analysis may involve over 41 lines and 23 angles to specifically locate the displaced vertebra’ [101]. John D Grostic invented a device called a cephalometer that measures the patient’s head, and the measurements were then used in conjunction with the radiographs [102]. Blair employed what he called a ‘protracto clamp’ and special chair to help position the patient for the radiographs [103]. Cowin placed small metal pieces on various anatomical landmarks on the patient’s head before x-raying, in order to facilitate subluxation analysis [82]. Some of these techniques used machines to perform the adjustments or employed other tools of questionable diagnostic utility, such as temperature sensing devices to augment the indoctrination process, but only x-ray-related machinery is considered in this paper. These ‘sacred artefacts’ helped the practitioner rationalise the system and engaged the patients into believing.

#### Sacred stories

Sacred stories form an important part of religions. They help capture peoples’ imaginations. They also take concepts that contravene the majority of human experience and work to elevate either a figure within the religion or the ideas of the religion itself. The idea of Christ walking on water makes him supernatural, inspiring awe and respect; it sets him apart, with abilities above those of mere mortals. It is then not unreasonable to believe that such a superior being should have his words listened to and obeyed. The story of the Buddha’s life path of asceticism and respect for all living creatures, as well as his ability to overcome challenges creates a desire to emulate the methods, to become part of the group that enjoys this way of life. Stories create a reality of the author’s imagination; they may inspire by showing what happens and why, giving a new perspective on the events in life, and can help listeners/readers understand, evaluate and redirect their lives [104]. The prototypical chiropractic sacred story is that of DD Palmer’s first adjustment, which he believed cured deafness in a man. DD then recounted this story as the breakthrough moment when he realised the principle of chiropractic. This would have been a persuasive tale in the days when monistic theories of disease were given serious consideration. In the current context, sacred stories were considered to be the tales of the diagnostic and healing powers of the technique (patient testimonials) or positive statements from practitioners who adopted the technique and believed that their practices flourished because of it (practitioner testimonials).

Typical patient testimonials included the following: ‘[The chiropractor] took x-rays, and my life was changed in about 2/3 of a second… My spine, which had been 20 degrees to the right, suddenly was realigned. One minute after he actually used the [adjusting] device on me, we took another x-ray … and it was the first time in twelve years that I was not in pain.’ (Atlas Orthogonality) [105] ‘He studies x-rays and makes precise and thoughtful recommendations. His pricing is reasonable. I love my body feeling good and highly recommend getting an adjustment if needed. You will be pleased…’ (Duff) [106].

Practitioners gave testimonials such as: ‘Patients know how long their correction-rehabilitative care will take and how much it will cost, making my pre-pays extraordinary!’ (Pettibon) [107]. ‘This [Advanced Orthogonal] seminar has been the most influential seminar of my life. It has changed not only the course of my career, but also the life of my family’ [108].

Case reports, a weak form of clinical evidence, [109,110] were considered sacred stories as well as case anecdotes. Case anecdotes were stories of patient benefit from the perspective of the practitioner, similar to case reports, but without scholarly rigour in the reporting. These were much more frequently cited in the publicly available documents of the technique systems studied here than higher levels of scientific evidence such as randomised controlled trials. The case reports and anecdotes were usually found in magazines or newsletters, rather than peer-reviewed, scholarly journals.

Unsupported extrapolations and oversimplified analogies of anatomy or physiology were also considered sacred stories. These were interpreted as the equivalents of myths or fables, conveying the morality and correctness of a particular system. The technique systems examined here warned of the detrimental effects of spinal subluxations. Some created analogies to other disease processes, which may become far advanced without the person knowing, until one day it is too late. For instance, the Blair system used several intricate, almost indecipherable analogies, claiming that the body has a momentum like a spinning fan blade [111]. Once a trauma creates a subluxation in the spine, disease begins in one cell then spreads to others geometrically [111]. ‘The longer the subluxation exists, the greater becomes the momentum of speed of diseases’ [111]. Kale conveyed the danger of spinal misalignment in the most extreme way: ‘Mild to moderate malfunction results in sickness and disease, while extreme malfunction results in DEATH!!’ [emphasis in original] [97]. These ideas have not been supported by evidence available in peer-reviewed literature [20,21]. They are likely useful to the technique, though, to help hold together its concept of disease, and to gain new believers in patients and practitioners.

Several of the techniques also related the stories of the way in which their founders either came to chiropractic or invented their particular system. William Blair claimed to have been cured of lifelong asthma by upper cervical adjustments [112]. Clarence Gonstead credited being cured of rheumatoid arthritis by a chiropractor when he was a child; receiving regular adjustments so invigorated him that he went on to maintain a superhuman practice schedule for years, apparently seeing 250 patients per day [75]. John F Grostic claimed to be cured of Hodgkin’s lymphoma, twice, by BJ Palmer himself [66]. Once healthy, he apparently also kept a gruelling practice schedule [66]. Sharon Freese-Pettibon (wife of technique founder Burl Pettibon) was evidently relieved of several life-threatening conditions, including paralysis, by her future husband’s ministrations [113].

Sacred stories also included distorted evidence to support the belief in the use of radiography: ‘In CBP® Technique, the use of initial and follow-up spinal x-rays or radiographs is deemed necessary; however, some in chiropractic have condemned the use of follow-radiographs to collect alignment data. Importantly, there is data [sic] to show that the use of medical/chiropractic x-rays constitutes a very minor health risk and in fact has been shown to be of benefit (decreased sickness and cancer mortality rates) in some studies’ [114]. Unfortunately, the studies cited either did not actually support the claim of benefit in that they either did not include humans as study subjects, or they did not study diagnostic radiation. The concept of radiation hormesis (beneficial effects) has been discredited [115]. The National Academies of Science have done a long-term, comprehensive review of all known types of radiation, called the Biological Effects of Ionizing Radiation (BEIR) studies. This is the best available evidence, from a consensus of a large majority of scientists, and indicated that there is no such thing as a harmless dose of radiation. Every dose has the potential to cause damage and doses are cumulative: ‘The report concludes that the preponderance of information indicates that there will be some risk, even at low doses, although the risk is small’ [116]. Given this information, CBP’s claims were considered a sacred story, used to support the radiographic paradigm of their system.

These stories may have performed the same function as myths and fables in religion. That is, they elevated the status of the authorities, they inspired awe and a desire to be part of the group, and they formed powerful tools for the recruitment of a flock (patients) and new shepherds (practitioners). See Appendix 4 for examples of the sacred stories of all the techniques.

#### Special language

Special language is used to describe unique or unusual artefacts, processes or rituals in various religions. It includes words like sacrament, reincarnation, and exorcism. L Ron Hubbard created an extensive vocabulary and copious acronyms for Scientology. For instance, ‘thetan’ represented a human soul, ‘enturbulated’ meant troubled, and SP stood for Suppressive Person, that is, someone who disapproved of Scientology [117]. These words seem to reinforce the exceptional nature of the religions and help make them more mysterious and worthy of wonder. In the realm of healthcare, Fuller observed that ‘A distinguishing character of these [unorthodox medical] systems is that they utilize vocabularies and techniques designed to induct individuals into a world view predicated upon the “fact” that under certain conditions extramundane forces enter into, and exert sanative influences upon, the human realm. They are, therefore, substantively religious…’ [118].

Special language used by the technique systems studied in this paper seemed to be an example of Fuller’s idea. The language found in the techniques’ descriptions of their radiographic processes included original words or specially derived meanings of ordinary or medical words. For instance, the word ‘subluxation’ has a meaning in medical literature that is different from the special definition given to it by subluxation-based chiropractors. Since the late 17th century, the term subluxation has denoted a partial dislocation of a joint, and implied severe damage to ligaments, tendons, and the other soft tissue structures that support articulations in the body [119]. However, the writings examined for this paper used the term to indicate a minute misalignment of a joint, sometimes claimed to be less than a degree of angulation or a sub-millimetric displacement [82]. These techniques also included in the definition a component indicating that despite the slightness of the lesion, nerves or the spinal cord were affected to a degree that created manifestations of disease in the organs of the body. Gonstead [120], Duff [121], Zimmerman [56], and others made statements to this effect.

Other special words are names of tools, such as the cephalometer mentioned above [102] and the anatometer, a device by Ralph Gregory of NUCCA, [122] used to examine posture in conjunction with radiographs to find subluxations. The term ‘orthogonal’ (which is a geometry term meaning ‘having right angles’) as applied by the different systems to the upper cervical spine, is special to those techniques, essentially denoting the ‘normal’ position of vertebrae. Blair used the term ‘derinothermographic’, undefined in their writings and not comprehensible by context: ‘The presence of cervical nerve interference is established by observation of both persistent differential paraspinal derinothermographic pattern…’ [103] The language of Chiropractic BioPhysics is so special that it protects its name and other terms such as PostureRay [123] with the® registered trademark symbol. Finally, several upper cervical techniques have radiographic views such as the ‘nasium’, ‘base-posterior’, and ‘vertex’, which are used for finding subluxations, and are not found in mainstream radiographic positioning texts.

Combinations of words in unique syntax are also part of the special language used by religions as well as these chiropractic techniques. The words ‘holy’ and ‘see’ are commonly understood separately, but when joined and capitalised, Holy See means the ecclesiastical jurisdiction of the Catholic Church in Rome. Nirvana is a word purloined into common English usage, but with the specific meaning of withdrawal from the cycle of rebirth to a Buddhist. In the world of radiographic detection of chiropractic subluxations, some genuinely unique usage of language was developed. Ronald Aragona introduced difficult to decipher definitions of his methods in Applied Spinal Biomechanical Engineering. For example, ‘bilateral symmetrical function is secondary to static non-dynamic gravitational dependent equilibrium’ [83]. Gonstead developed a complex system for describing subluxations on radiographs [124]. Kale also made statements thick with jargon: ‘If the apparent listing does not clear the scan, then the listing will be re-evaluated and changed until the end result is a clear pattern on the scan’ [125]. Special language was seen to help define each system, differentiating it from similar systems, and in the case of the techniques that use radiography for subluxation detection, this language may have given patients and practitioners a feeling of being involved in a special group with superior health knowledge to mainstream medical and chiropractic practitioners. See Appendix 5 for additional special language in the technique systems.

### Historical links

These findings indicate that a system of belief has operated, and is still present in parts of the chiropractic profession. This belief system demonstrated qualities in common with religion rather than science and a reliance on clinical evidence. This is not to say that these techniques viewed themselves as religions, or that religion was a consciously embedded component during their creation. In fact, quite the opposite was frequently claimed; these techniques often stated that they were scientific [57,83,113,126–129]. Science, though, as has been noted, had a unique definition within some circles of chiropractic [130]. If technique systems displayed characteristics of religion rather than science in their stated application of radiography, an important question is raised. Why would this be so? In addition to the justifications for radiography discussed in the Background section above, there seemed to be a historical connection to early chiropractic justification for radiography. That is, the rationale for the use of the x-ray as given by BJ Palmer seems to have been adopted by technique systems subsequently developed throughout the 20th and into the 21st centuries. Evidence for those historical links will now be elucidated.

First, consideration should be given to the trial of Shegataro Morikubo and how it shaped chiropractic. In 1907, Morikubo was arrested for practicing medicine without a licence. Attorney Tom Morris successfully defended him. Morris invoked the concept that chiropractic was ‘separate and distinct’ from medicine and osteopathy by reason of chiropractic’s unique philosophy, science and art [131]. For the most part, he used evidence of the workings of the profession from Solon Langworthy. Langworthy had been trying to advance the cause of modern science in the profession and was strongly opposed by both BJ and DD Palmer [131]. In Morikubo’s defence, Morris referred to Langworthy’s assertion that the brain was a source of ‘unseen power’ in the body, transmitting this power through the nerves to the organs, which could be blocked by out-of-place vertebrae [131]. For an unknown reason, Morris did not call Langworthy as a witness. However, BJ Palmer testified, and supported this refinement of the idea of chiropractic then and in many subsequent trials [131]. It is from this experience that Palmer adopted Langworthy’s definition of ‘subluxation’, the concept of the brain supplying life force through the nerves, which he then fervently promoted [131,132]. So although the emphasis on, and singular importance of, the chiropractic subluxation to general health did not originate with BJ Palmer, Palmer’s preeminent influence on the profession until at least 1925 was undeniable [131,133]. Palmer was the major source of the dissemination of these concepts, which he saw the opportunity to reinforce with the advent of diagnostic x-ray technology.

Perhaps additional insight was given by Wilson in his book *Spinatology* (1955), [134] in which he indicated the fear of insignificance as well as the desire to be thought to be working for good:

Why discuss the question of an inborn intelligence? First, if there were no inborn intelligence, Chiropractic would not make sense. Secondly, and of no little importance, it gives us a feeling of working with a higher power, in which we are helping to make more complete its expression in the human body. Such an idea certainly adds to the dignity of our work and causes us to feel more important. Then we can recognize more fully the real value of our work for suffering humanity [134].

The second argument seems to be simply a longing for belief in the unseen, and the circularity of the first argument is obvious: inborn intelligence (Innate) must exist, otherwise chiropractic cannot exist; therefore Innate must exist because chiropractic exists.

Cyrus Lerner was an attorney commissioned to examine the chiropractic profession with a view to improving its image in the mid-20th century [135]. He produced a report realistically portraying what he found, and the report was buried [136]. In it he noted that chiropractic offered ‘highly questionable evidence’ regarding ‘nerve impulses’ and that ‘Nearly all the writings examined on the “Story of Chiropractic” gave the feeling of one wandering through a fog’ [135]. Perhaps it is not surprising that, building on this foundation, some descendant practitioners demonstrated similar thinking. The public documents examined for this paper indicated that observations and modifications of methods were limited to improving the description or quantification of subluxation radiographically. There was no evidence found of exploration as to how misaligned vertebrae actually caused ill health. As Keating [2] noted, ‘BJ’s “science” was constrained to support the “truths” of chiropractic that he was promoting’ [2].

In discussing cults within chiropractic, CO Watkins, himself a chiropractor, noted:

Each has accepted a particular system with the same finality that one accepts his religion, and assumes that its methods represent the alpha and omega of chiropractic knowledge. They make no attempt to review other knowledge, to test their own methods for specific end results, or to compare their methods with others. They develop a faith in their methods which precludes any attempt to examine others; the same attitude of faith removes any necessity in their opinion of critically examining their own methods. Their approach to the patient is that of the cultist. They seek through philosophy and logic to instill in the public their own faith in their methods, at the same time tyring to discredit all other methods of healing the patient may have confidence in [137].

To call these techniques cults may be hyperbolic. But Watkins’ observations seem to have a certain merit, at least as represented by systems the examined for this paper.

Two of the characteristics of religion discussed here, claims of supremacy to other techniques and the sacred stories of testimonials could be viewed simply as efforts to obtain business advantage. It is certainly possible that some of the techniques use them in that way. However, the overall context of the writings of these systems conveys a different message. It may be legitimate to view them as religious, or at least bearing a great resemblance to religion. The mysteries of the universe have led people to invent ideas to explain them when they could not yet discover the real explanations. So, too, the mysteries of the human body’s structure, function and dysfunction have led people to invent explanations for the as yet inexplicable processes of disease and health. A religion-like thread is visible, woven into the contemplation of such matters, from the earliest days of healthcare development in the United States. Gaucher-Peslherbe noted that in ‘the rationalisations that the [theories of healthcare] were based on were often no more than articles of faith which it was heresy to renounce’ [40]. Gaucher-Peslherbe quoted Bernard, who also saw evidence of the quasi-religious in non-mainstream health practitioners, as well as a vulnerability in the public at large: ‘Believing only in himself and rejecting all authority the empiric can boast of anything he likes, and claim that he can cure all ills. He will always find enough people who believe him to encourage him in his quackery, for men are so made that they have a need to be deceived; the miraculous is preferred to the real, and people would rather believe in inspired than acquired knowledge’ [40]. Certainly the path to that belief is streamlined with the modern ‘miracles’ of technology, like the x-ray to show the subluxation as the cause of disease. Perhaps the subluxation is imaginary, perhaps not, but that makes the radiograph no less powerful a talisman.

Wright [117] noted that it is one thing to create a plausible universe, but quite another to believe it. ‘That is the difference between art and religion’ [117]. There was no evidence of art, or hucksterism, found. All the advocates of the various techniques seemed sincere in their belief of the usefulness of the radiograph in demonstrating a tiny, but life-endangering misalignment of bones. The friction arises in that health care systems should not be based in belief, but in evidence. They should not be dogmatic, but adaptable to new information. They should not be self-centred, but patient-centred. As Phillips and Mootz noted: ‘A complete reliance on a holistic universal intelligence entails dogma and is not acceptable in current chiropractic philosophy or practice’ [36].

Fuller [138] observed that a sense of wonder was a principal source of belief in the existence of an unseen order of life [138]. Certainly a personal ‘miracle’, in this case a cure by unconventional methods, after a period of strife and disillusionment with conventional methods, would engender a sense of wonder. Then the unseen order of life is revealed by the chiropractor providing the treatment, and the patient becomes a believer. Once belief is entrenched, it is exceedingly difficult to change, even when the believer confronted with incontrovertible evidence [139]. So these practitioners’ explorations within their beliefs, without questioning the core principles of them seems explicable. But is it acceptable within the profession? Can these methods thrive in a world moving towards evidence-based healthcare?

The idea of healthcare ritual as being similar to religious ritual is not restricted to chiropractic. Welch [140] noted that due to their unique powers to see into human beings, to understand how we functioned and what had gone awry, ‘before the priest and physician, we stand individually transparent’ [140]. The healing powers of pre-medical practitioners have been partially attributed to ritual and the placebo effect that it brings [140]. A full discussion of this subject is beyond the scope of this paper, but from the evidence gathered herein, it does seem plausible that the ritual of radiography may have played some role in creating the successes claimed by the practitioners.

It may be argued that medical artefacts, such as the white coat, the stethoscope, and the scalpel might also be construed as sacred, that the trappings of any profession create an atmosphere conducive to indoctrination. The difference is that medical writings do not refer to vital energy, or a single cause of disease, or dis-ease. They do not advertise that once magnetic resonance imaging reveals a tumour, subsequently removed by the surgeon, the flow of life force will be restored and other, unrelated ailments may then be healed. The intent of the chiropractor, who uses his or her superior knowledge and the reputation of a learned profession to persuade, cajole, or frighten lay people into belief in a particular system is the differentiating factor. The overall impression from studying the chiropractic systems in this paper was that the intent of the documents was to win new converts by indoctrination. It did not give the impression of educating the public to choose a healthier lifestyle by fully informing themselves of physiological and pathological processes as best understood by science. The tone seemed to be one of conspiracy. The implication was that the practitioner knew a secret to health that was somehow beyond the grasp of mainstream medicine, or perhaps suppressed by it. The idea of limiting healthcare to a single cause, to be fully understood only by one man, the originator of a technique system, transformed into saviour because of his understanding, was a theme that emerged from the characteristics of each system. This environment, with all the characteristics taken together, their denotations and connotations, is what evoked the idea of religion rather than science.

Religion seems to limit the possibilities of the way the world works. By accepting one religion and its views on the machinations of the universe, one must necessarily discount other views, limiting perspective to a certain framework. Life becomes easier. It is satisfying to be part of a community and to be told that the complex, confusing world is actually simple and explicable. This is one of the psychological benefits of religion [141,142]. The chiropractic systems that use radiography for subluxation detection limit the possibilities of the way health and disease work. The claims of supremacy that each technique espouses would seem to indicate that the practitioners thereof derive satisfaction from their understanding of health. However, they limit the potential benefits that could be achieved for their patients if less partisan, more objective research was employed to discover the mechanism of action of chiropractic methods.

#### Limitations of the study

It is possible that some techniques were inadvertently excluded if they no longer exist, had very small numbers of practitioners, and/or no internet presence. This paper only sampled the publicly available writings from techniques’ official websites, papers, and textbooks; nuances and variations would likely be revealed by interviews with the spokespeople for the techniques, for those techniques that are not defunct. Also, because websites are sometimes created or edited by external individuals or companies conversant with information technology but not necessarily the subjects of the sites they design, they may not always accurately reflect information presented to them for publication. Differences would also likely have been found if individual practitioners were interviewed on how they practically applied the techniques in their own practices, rather than just how the techniques officially advocated use of their systems.

### Summary

The founders and early pioneers of chiropractic did not benefit from the current understanding of science and research and therefore substituted deductive and inductive reasoning to arrive at conclusions about health and disease in the human body. Some of this thinking and rationalisation demonstrably followed a religion-like pattern, including BJ Palmer’s use of radiography. Access to scientific methods and research education became much advanced and more accessible during the past few decades. However, the publicly available documents of technique systems that used radiography for chiropractic subluxation detection employed a historically derived paradigm for radiography that displayed characteristics in common with religion.

## Appendix 1: evidence of supernatural concepts in the technique systems

### Advanced orthogonal

Vitalism and supernatural calling: ‘By God’s design, the balanced function of the brain and nervous system is essential to the integrity of health. Our focus is on identifying and correcting the structural causes of this dysfunction, to restore and maintain the health and well-being of all those whom we serve. Too many people are suffering from conditions that are impeding their ability to live fruitful and productive lives. We want to let these people know that there is hope for answers, and opportunities for healing. Finally, we recognize that the human body is fearfully and wonderfully made, and our desire is to honor the Creator of this dynamic body through our approach to health care’ [72].

### Applied spinal biomechanical engineering

Vitalism: ‘A chiropractic doctor detects and corrects these spinal misalignments (vertebral subluxation causing nerve interference), restoring normal nerve impulses to the body allowing the body's own innate healing capabilities to function optimally’ [67].

### Applied upper cervical biomechanics

AUCB only obliquely references vitalism with phrases like ‘neural reintegration’ and they are couched in science-like terminology. However, the original chiropractic premise is implied by statements like the following: ‘The foundation of the chiropractic profession stands upon the premise that health and disease are nervous system dependent,’ ‘How can I transcend the practice of pain management to the practice of true health care? We all have been told that chiropractic can effect [sic] the body as a whole, but where is this practice? These are the questions our association is striving to provide objective answers to’, and ‘If you are ready for a practice that delivers consistent broad-scope health care, then welcome to the IUCCA [International Upper Cervical Chiropractic Association]’ [143].

### Atlas orthogonality

Vitalism: ‘When the atlas bone is properly aligned, that is in the orthogonal—or neutral—position, the rest of the spinal vertebra [sic] come into better alignment allowing the body to heal itself’ [144].

### Blair

Vitalism: ‘The nervous system controls and regulates all parts and functions in the body. When there is nerve interference (subluxation) your body loses the ability to properly self regulate and heal itself. The result can be pain and illness. Blair upper cervical care is often the key to people regaining and maintaining good health’ [69].

Supernatural calling: ‘There is a verse in the Bible- Chapter 9, Verse 6 in II Corinthians… by changing a few words, we can make this revealing verse read - "He which soweth competence and precision will reap outstanding results, and he which soweth carelessness and inaccuracy will reap guilt and mediocre results”’ [112].

### Chiropractic BioPhysics

Chiropractic BioPhysics (CBP) makes no explicit references to religion or vitalism however, however their stated goal is to ‘correct human spines and alleviate human suffering’ [70]. CBP also aligns itself with the International Chiropractic Association (ICA) and World Chiropractic Alliance (WCA) [145].

### Cowin upper cervical orthogonal

Vitalism: ‘Our procedures are based on the traditional chiropractic concept of vertebral subluxation’ [68].

### Duff

Vitalism: ‘The goal of a Chiropractor is to correct the "Vertebral Subluxations" (misaligned vertebra the cause a disturbance to the nervous system and other systems) for the purpose of restoring the proper communication of nerve energy over nerve pathways that exit the spine’ [121]. ‘Most of this problem (Subluxation) [sic] exists at the base of the skull or known as the upper cervical region of the spine. It is at this level of the spine that can produce many health problems and is where I often first focus my attention, even if there are lower spine problems, This is because of the likely hood [sic] of Upper Cervical [sic] involvement. It's importance cannot be overlooked both in terms of preve*ntion* [sic] of sickness or disease or correction of dysfunction of the body and its return to optimal health’ [121]. Duff’s logo is a chiropractic angel, which is common throughout the profession, but with the addition of radiating lines that make it look particularly heavenly [77].

### Gonstead

Vitalism: ‘…compressed nerves often become inflamed and impede the proper transmission of impulses to the section of the body controlled by these nerves. A seemingly endless list of ailments and pain may be brought about by these subluxations’ [120]. ‘It is the goal of your Gonstead doctor to restore and maintain optimal health by locating and correcting any interference to the nervous system caused by vertebral subluxation’ [146]. The picture on the official website shows Gonstead showing him with hands clasped, wearing a white shirt with high, priestly collar, staring beatifically into the distance, a halo emanating from his upper body [75].

Supernatural calling: ‘Our Mission is to perpetuate the teachings of Dr. Clarence S. Gonstead, fund chiropractic research, and encourage cooperation and camaraderie amongst all who practice the Gonstead technique’ [73]. ‘To spread the word about the Gonstead technique, we need to be in the public eye. You can help’ [147]. Gonstead chose for his clinic the town of Mount Horeb, [75] named after the Biblical site of Moses’ receipt of the Ten Commandments.

### Grostic

Vitalism: ‘When the vertebrae become misaligned (subluxated) a decrease of innate vital energy between the brain and the body occurs causing malfunction and dis-ease. Chiropractors release the innate power of the body within by detecting and correcting spinal subluxations’ [148]. ‘…misaligned vertebra(e) impinge the flow of innate intelligence and cause illness, or what BJ Palmer later referred to as dis-ease [66]. ‘Orthogonally-based upper cervical care is not a treatment for conditions or diseases, however, this subluxation-centered care has been shown to have an associative effect on various conditions’ [149]. Christian religion was also directly injected into Grostic seminars, as each day began with a prayer [66].

### Kale

Vitalism: ‘When one or both of the top two bones (Atlas and Axis) misalign, creating pressure on the "Brain Stem" and causes an interference of nerve flow to all parts of the body. This is what causes the body organs and tissues to malfunction, thus causing disease’ [127]. ‘The "Specific Chiropractor" ("Brain Stem Specialist") is trained to locate and remove this subluxation (misalignment) and restore the vertebrae to its normal position. The mental impulse, life force, Innate Intelligence, can flow in its full capacity from the "Brain Stem" to its diseased tissue or organ; healing or repair takes place in a natural way and pain and the ravenges [sic] of disease disappear’ [97]. ‘Mild to moderate [spinal] malfunction results in sickness and disease, while extreme malfunction results in DEATH!!’ [emphasis original] [97].

Supernatural calling: ‘The Kale Foreign Messenger has been in over 9 countries doing missionary work’ [74].

### Knee chest upper cervical specific

Vitalism: ‘**Upper Cervical Specific Chiropractic Care** focuses on removing nerve irritation to restore communication between the brain and the body. Restoring function at the level of the brainstem and upper cervical spine (the control center of the body) is essential so that the body may heal itself and have optimal vitality. With the nervous system functioning at 100% the body has the potential to heal itself from even the most complex of ailments’ [127]. ‘a significant academic influence running through every KCUCS module. KCUCS finds a good balance between “bringing the Spiz” of Upper Cervical Chiropractic while also bringing the science’ [150]. Spiz or spizzerinctum was BJ Palmer’s word meaning enthusiasm for chiropractic.

### Logan basic

Vitalism: displacement of the sacrum is the root cause of disease. Claims of cures of various organic conditions such as asthma and appendicitis, with pre- and post-treatment full-spine radiographs provided as evidence showing distorted and corrected vertebral and pelvic alignments [151].

### Mears

Vitalism: ‘The Mears Technique is a specific upper cervical system of X-ray analysis and adjustment that carries on where the H.I.O. concept ends’ [152].

### National upper cervical chiropractic association

Vitalism: ‘The postural rebalancing created by the upper cervical adjustment allows for the body to have less gravitational stress and therefore the person can direct that now unused energy to self-healing, maintenance and thinking’ [153].

### Orthospinology

Since Orthospinology is essentially inseparable from Grostic; the same vitalistic concepts apply [66].

Supernatural calling: ‘I encourage each of you to thank God… for the job that you have… Correcting an atlas misalignment and restoring your patient’s body balance, integrity, and health…’ [86].

### Palmer upper cervical specific (Hole-in-One)

Vitalism: ‘That the *sunnum bonum* of all life and death, health or disease issues pivoted around a study of the correct or incorrect position of vertebrae’ [39].

### Pettibon

Vitalism: ‘The purpose of the chiropractic adjustment is to work “with” the innate intelligence of the body to produce a predictable response. Our specific adjusting forces are designed to cause innate to react positively with its environment. An adjustment must be a positive, constructive force that causes the body to predictably react to its environment, correcting the spine so the nerves can function normally resulting in health to be expressed optimally’ [101].

### Pierce results

Vitalism: ‘By correcting subluxations more effectively, Dr. Pierce began to see many things heal faster, even spines that never changed on x-ray were now exhibiting complete corrections in minutes! (Thanks to Innate!)’ [154].

### Spinal orthopaedic neurological advancement and research

Vitalism: ‘God built within us the ability to heal ourselves. If the communication between the brain and body becomes disrupted, this ability degrades. The atlas is the top bone in the neck. It houses and protects the brainstem and upper spinal cord, which is the communication center between the brain and body. When the atlas misaligns, it irritates the delicate nerve tissue’ [155]. ‘The procedure cures nothing, but it allows the body to cure itself. It restores body balance and proper nerve flow so that organs, limbs, and tissues can resume normal functioning. This is why we see so many different conditions with no apparent relationship responding to the SONAR procedure’ [156]. ‘… [The] result of a misaligned atlas is the restriction or distortion of critical messages at the brainstem and upper-spinal cord, disrupting the brain's communication. Every cell, organ, or tissue that is not receiving adequate nerve energy and communication from the brain will suffer and degenerate’ [155].

Supernatural calling: ‘Through the grace of God, Dr. Elliott has developed the SONAR instrument’ [71].

### Spinal stressology

Vitalism: ‘When you put subluxations in terms of a disorganized system and the treatment of subluxations as you are trying to release that pattern of disorganization and bring it back into a reorganized format, where it can recover and create its own homeostasis…’ [128]. ‘Is it possible to read a persons personality or emotional history by an analysis of their spinal alignment and posture? Although from a superficial standpoint it may seem obvious that a depressed person would carry himself differently than a happy one, we have found that a radiographic spine/posture analysis can reveal stresses that correlate to emotional trauma, deep-seated personality characteristics, inherited trauma and relationship issues’ [157].

### Sutter

Vitalism and supernatural calling: ‘That there is a Creative Intelligence in back of all laws and life on earth, call It God, Universal Intelligence or what you will. That we are instruments placed upon this earth for self-development and service… That health is natural and disease is unnatural. That disease is caused by an interference with the Creative Intelligence that wills normality within the body. That such interference is caused by obstruction of nerve impulse flow from the brain to the body due to a subluxated spinal vertebra. That this Chiropractic principle is the greatest boon to suffering humanity and supplies a need for which there is no substitute’ [129].

### Zimmerman

Vitalilsm: ‘All it takes to restore the flow of energy to the sick body is ONE accurate adjustment.’ [emphasis original] [56].

## Appendix 2: evidence of claims of supremacy to other methods

### Advanced orthogonal

- ‘…the most accurate assessment of spinal misalignment’ [158].
- ‘Today, Advanced Orthogonal chiropractic stands alone as the most evolved offspring of the upper cervical procedures’ [159].
- ‘Upper cervical doctors utilizing the Advanced Orthogonal have acquired that additional education’ [160].
- ‘With the increased accuracy of Orthogonal chiropractic, Advanced Orthogonal doctors have higher expectations for correcting patient subluxations’ [160].
- ‘Digital x-ray analysis to measure misalignments to the 1/100th of a degree’ [81].

### Applied spinal biomechanical engineering (ASBE)

- ‘…doctors discover how little they knew before they started learning these methods and procedures. With this scientific work incorporated in their clinical setting, a new enthusiasm manifests. Practitioners are no longer bored with methods and procedures. They are continuously learning and providing a better service to humanity’ [83].
- ‘…a dependable and proven effective procedure in improving patient’s posture that can be added to your current adjusting protocol’ [83].
- ASBE provides ‘…a remarkable advantage over other methods and procedures…’ [83]. They claim to base their methods on ‘…12 volumes of A.S.B.E. research discoveries’ [83]. These works seem to be self-published [67].

### Applied upper cervical biomechanics (AUCB)

- ‘Our association’s research into this critical area of the spine has founded a highly effective form of health care that is unique from any other in the world’ [161].
- Radiographic landmarks form the foundations of entire systems of analysis; hence their reliability is paramount to the accuracy of the evaluation [162].
- ‘…AUCB uses a complex and unique form of upper cervical radiographic analysis’ [84].
- ‘Adjustments based on this system of analysis have consistently produced full body neurophysiologic benefits on patients, which has been objectively substantiated by both high-resolution camera and paraspinal infrared imaging’ [84].
- ‘…15 years of unprecedented research…’ [84].

### Atlas orthogonality

- ‘Improving the health and extending the lives of people around the world’ [163].
- It also claims to provide certainty of diagnosis and treatment for the practitioner, ‘There is no guess work [sic] in this program’ [144].
- ‘…the most precise atlas correction…’ [144].

### Blair

- ‘This “blue print” of your neck [created from the x-rays] allows the Blair chiropractor to deliver the adjustment that is exactly what your body needs’ [69].
- Patients ‘enjoy better health, less pain, less illness and greater quality of life’ [69].
- ‘…[it’s] possible for a chiropractor to achieve excellent if not outstanding results. This chiropractor will eventually find that he will be able to make the spinographs and give the adjustment with confidence and ease’ [112].

### Chiropractic BioPhysics (CBP)

- ‘Chiropractic BioPhysics®, or CBP®, is, in short, a higher level of chiropractic. It is a more knowledgeable, comprehensive, systematic, scientific approach to chiropractic, that provides predictable results for patients and will contribute to building a more stable and successful chiropractic practice’ [126].
- This technique’s literature also appeals to the readers’ rational inclinations, ‘CBP® is the most published named chiropractic Technique [sic] within the Index Medicus in the history of Chiropractic. CBP® has more published papers in peer-reviewed indexed journals than all other techniques’ [164].
- Photo of a man tearing open his shirt to reveal a cape and the logo of a business-enhancement company underneath, associated with CBP [165,166].
- CBP® is ‘…the best technique in chiropractic’ [167].

### Cowin upper cervical orthogonal

- ‘Our methods were observed by Wollongong University mathematicians Aldis and Hill and written up in the Journal and Proceedings of the Royal Society of NSW in 1979’ [68].
- Measurements ‘as small as 0.75 degrees (or, translated into linear measurements, each as small as 28 thousandths of an inch or 0.7 mm)’ [82].

### Duff

- ‘Dr. Duff's speciality is in aligning the upper top two vertebrae Atlas Axis, C1 and C2 [sic] by using specific X-Ray views …’ [77].
- ‘…tailor-made, specific adjustment…’ [77].

### Gonstead

- ‘…why don’t all chiropractors use this technique? Because the analysis takes more time and mastering the art of delivering a specific adjustment takes a LOT of practice and dedication’ [emphasis original] [57].
- ‘…one of the most advanced and scientific methods [of correcting] spinal misalignments, joint dysfunctions and subluxation complexes’ [57].
- ‘The Gonstead Chiropractor goes beyond what many chiropractors consider a spinal assessment by conducting a thorough analysis of your spine using five criteria to detect the presence of the vertebral subluxation complex’ [57].
- ‘The focus of the Gonstead adjustment is to be as specific, precise and accurate as possible, addressing only the problem areas (areas of subluxation).’ [146]
- ‘There is a worldwide referral network of qualified Gonstead chiropractors using the same methods of analysis, the same specific adjusting procedures, and who share the same goal... to correct the subluxations in your spine for the purpose of creating a healthier you!’ [168].

### Grostic

- ‘[Grostic is a] method for analyzing and correcting the occipito-atlanto-axial subluxation complex. It is actually a series of steps in the total care of the patient and is therefore a chiropractic procedure and not simply a spinal adjusting technique’ [149].
- ‘The analysis of your x-ray views will reveal the exact degree of misalignment or imbalance. The mathematical measurements taken from your x-ray films give the doctor the correct formula to realign your spine’ [149].

### Kale

- The motto of the affiliated TIC (as in ChiropracTIC, from BJ Palmer) Institute is ‘Principled, Dedicated, Pure’ [169].
- ‘Dr. Kale was the world's leading authority on "Upper Cervical Specific" Chiropractic Knee-Chest Technique’ [74].
- ‘DO I NEED THIS PROCEDURE? Unlike knowing when you are hungry, or when you have to go to the bathroom you DON'T know if you are in SUBLUXATION. A Specific Chiropractor can tell you if and when you need this procedure, using specialized scientific scanning equipment’ [emphasis original] [127].
- ‘DOES THIS REALLY WORK? YES!!! For 100 years people with all kinds of sickness and diseases have been getting well from this procedure. YES!!! There is research to back up this procedure. YES!!! There are plenty of satisfied patients, over 40 million.’ [emphasis original] [127].
- ‘This new relationship brings the legacy of Dr. Michael Kale through his son, Dr. B.J. Kale to The Specific Chiropractic Center. Dr. Michael Kale was responsible for the perpetuation of the Knee Chest Upper Cervical Specific work during a vital time in the history of Upper Cervical Specific Chiropractic. Dr. Michael Kale was the only Associate of Dr. Lyle Sherman, who was the Assistant Director of the BJ Palmer Research Center, prior to launching his career as a noted healer and disciplined teacher. His legacy extends throughout the world, having trained almost every noted authority in the Knee Chest Upper Cervical Specific world and having provided care to citizens of numerous countries around the world.
  Today, the tradition of the Kale name is carried forth by his son, Dr. B.J. Kale, who continues to teach around the world and uphold the values instilled in him by his father through The Kale Chiropractic Research Clinic, The Kale Foundation and the Kale teachings at the TIC Institute, an in depth training program for Doctors and Students who desire to learn the methods of his father, and the principled deliverance of the Knee Chest work through the BJ Palmer Research years of 1935-1951 under the direction of Dr. Sherman and Dr. B.J. Palmer. The Kale Chiropractic Research Clinic and Kale Foundation will continue to provide care to the people around the globe’ [170].

### Knee-chest upper cervical specific

- 6 week ‘X-ray Bootcamp’ [171].
- ‘The misaligned segments are identified through the use of Digital Laser-aligned Radiography. Because the misalignment is very slight (measured in millimeters) it is very important that precise x-ray equipment is used’ [127].

### Logan basic

- ‘…the secret of health and longevity has been solved… in Logan Basic Technique more than by all other asserted advances in healing methods since the beginning of time…’ [85].

### Mears

- ‘…more reliable points of reference to determine cervical distortion more accurately. This in turn led to the need to modify the adjusting procedure used in the H.I.O. technique’ [152].

### National upper cervical chiropractic association (NUCCA)

- ‘NUCCA has provided the chiropractic profession with more biomechanical data than any other chiropractic entity concerning the atlas subluxation, its effects on the spinal column and human body, how to restore its misalignments to the vertical axis, and has shown acceptable and measurable proof of the benefits of the chiropractic adjustment on the human body’ [102].
- ‘Defining the basic “problem” in chiropractic has been in dispute since the beginning of the profession. Measuring a “bone out of place” has eluded the vast majority in Chiropractic. While the rest of the profession has essentially walked away from this difficult concept, upper cervical chiropractic has been quietly and accurately measuring and correcting upper cervical misalignments (subluxations) for more than sixty years’ [172].
- ‘NUCCA’s forte, even among the other upper cervical specific groups, has been to deal directly with postural imbalance. Indications when to and when not to intervene are dependent upon postural measurements. The direction of vectored manipulation, the how to intervene, is dependant [sic] on X-ray analysis and an understanding of biomechanics and patient abnormalities’ [122].
- ‘NUCCA practitioners in general appear to have a more rigorous system with more pervasive and refined posture measurements, more meticulous X-ray procedures, and consistently better corrections as evidenced by its certification process and standards’ [122].
- ‘Gregory was able to independently develop or improve upon both the accuracy and precision of all levels of analysis of the “Grostic” system by 1) development of a double-pivot-point system in X-ray analysis (1962)… 5) design of better film analytical instruments… 7) design and development of the Anatometer which reduces the need for X-rays (1978), 8) classification of C-1 subluxations into basic types(1980)…’ [122].

### Orthospinology

- ‘…one of the greatest health care procedures in the world’ [173].
- ‘…has helped millions of people overcome countless health problems’ [173].
- ‘…greatest health care procedure in the world’ [86].
- ‘…revolutionary advances by Dr. John F. Grostic to upper cervical chiropractic care in detecting, quantifying, and correcting the upper cervical subluxation complex. All of these events have led to what is known as orthospinology and orthogonally based upper cervical care’ [66].

### Palmer upper cervical specific (HIO, toggle recoil)

- BJ Palmer spent much of his time in self-promotion; examples are replete throughout his writings and speeches [2].

### Pettibon

- ‘In 1988, Dr. Pettibon was honored for his work as the recipient of the Daniel David Chiropractic Scientific Award’ [113].
- ‘We have an unwavering passion to help you become the best practitioner that you can be. Our goal is to enable you and your staff to reach new levels of success in delivering patient care while simultaneously maximizing the efficiency and profitability of your clinic’ [113].
- ‘Dozens of doctors, on the verge of leaving the profession, have reclaimed their calling because of the understanding and protocols which is now known as The Pettibon System’ [113].
- ‘Most Doctors of Chiropractic laugh at the idea of "hit and hope" or "pop and pray" chiropractic procedures. Unfortunately in the “dark hours” of being alone, they wonder if what they are doing really works. They desire technical certainty. This is what Pettibon Spinal Biomechanics explains’ [101].
- ‘Conventional chiropractic x-ray procedures don't consider spinal soft tissue injuries. The Pettibon System's x-ray procedures do’ [100].
- ‘The Pettibon Procedures are an advanced, scientific approach to Chiropractic…’ [101].

### Pierce results

- ‘not a chiropractic technique like any others, but a systematic approach to correcting subluxations using the most advanced tools for spinal analysis and adjusting [154].
- ‘consistent and predictable results’ [154].
- ‘By correcting subluxations more effectively… even spines that never changed on x-ray were now exhibiting complete corrections in minutes!’ [154].
- ‘We don’t simply claim to provide the best chiropractic has to offer… we can back it up!’ [154].

### Spinal orthopedic neurological advancement and research (SONAR)

- ‘…far superior to other upper cervical techniques’ [71].
- ‘…[regain] health and wholeness without polluting your body with drugs and chemicals…’ [156].
- ‘…we have patients who come in from around the country to receive upper cervical chiropractic care’ [174].

### Spinal stressology

- ‘I'm kind of a revolutionary scientist…’ [128].
- ‘…when you are introducing something new (Thomas Kuhn has a book on it, called The Structure of Scientific Revolutions) there is always incredible resistance because you can't take the current concepts and explain the revolutionary concepts. You have to go with the revolutionary concepts and you almost have to unlearn so many things that you have learned to understand the new concept’ [128].
- ‘…we have documented more chiropractic cases than any before’ [128].
- ‘I've been doing this for 25 years’ [128].
- ‘Dr. Stephen Ward, an experienced Long Beach chiropractor, has a unique approach to pain management and treatment. He pioneered the use of the wall adjustment technique to provide more effective, longer-lasting adjustments’ [175].
- The official website provides a link to a video of an animated male figure in red and blue tights called ChiroMan doing a dance and some muscle stretches to heavy metal music [176].

### Sutter

- ‘Specific Atlas Correction deals with the specific location and correction of the cause of such interference within the nervous mechanism. It represents the latest development of scientific exactitude in the location and correction of the cause of disease within the body’ [129].
- ‘The exact variation of the atlas subluxation in relation to its surrounding structures peculiar to the individual case must be accurately determined. This can only be done through a specialized X-ray technique and analysis’ [177].

### Zimmerman

- ‘Due to the precision of the x-rays and other methods, ‘[the chiropractor] is trained to place the tip of the machine so accurately as to do that which has never been done before’ [56].
- ‘…opens up a field for practice with absolutely no competition’ [56].

## Appendix 3: evidence for the rules and rituals

The examples cited below are specifications or additions to the findings that all the technique systems had the rule that chiropractic subluxations were visible on radiographs, and all included the ritual of taking the radiographs as well as analysing them for subluxations.

### Advanced orthogonal

- ‘…consistent clinical procedure for accurate evaluation and reduction of the atlas subluxation complex’ [159].
- ‘This assessment can only be obtained by looking at very precise x-rays from all 3 dimensions. The views are left (sagittal or side), center (nasium or front) and right taken from above (vertex)’ [160].

### Applied spinal biomechanical engineering

- ‘ASBE utilizes very low radiation dosage static and dynamic spinal radiographs to help with objective pre-treatment and post-treatment diagnostic rational [sic] for the reduction of vertebral subluxations…’ [83 p. 2]. ‘ASBE starts the analysis with the static weight bearing x-ray views. Then adds intersegmental bilateral lateral flexion biomechanical analysis’ [83 p. 2].

### Applied upper-cervical biomechanics

- Radiographs must be obtained using the same views for every patient and that they are analysed the same way every time [178].

### Atlas orthogonality

- ‘The first step of the diagnosis is to determine the degree in which the atlas is misaligned. Very precise x-rays are taken which show the doctor exactly how the atlas is displaced. The x-rays taken are invaluable in making the most precise atlas correction which is as unique to the patient as their own fingerprint’ [144].
- ‘One of the most important post adjustment steps are post x-rays, which are taken immediately after the initial treatments… These three dimensional x-rays are taken and analyzed to verify that the best possible correction was rendered. The doctor may then show the patient the comparison between before and after x-rays. Post x-rays are an illustration of the exact change that has taken place because of the atlas orthogonal correction’ [144].

### Blair

- ‘Precise x-rays are taken to custom tailor the neck adjustment to your individual spinal anatomy and condition’ [69].
- These x-rays are ‘the hallmark of the Blair Upper Cervical Technique’ [179].

### Chiropractic BioPhysics

- Radiographs are obtained for every patient and analysed in the same way every time, obtaining post-treatment radiographs to verify changes but, uniquely to CBP, comparisons are made to ‘ideal’ posture calculated with a geometrical formula [180].
- ‘CBP® protocols require that the doctor must measure the displacements on spinal radiographs (segmental Subluxation). Both lateral-side view and anterior to posterior (AP) or frontal view CBP® x-ray line drawing procedures [are necessary] …’ [114]. and ‘…CBP® utilizes standardized x-ray positioning procedures…’ [114].
- Ease in applying the rules is available to the computer-savvy with CBPs proprietary x-ray line-drawing software, called PostureRay: ‘Sick and Tired [sic] of marking [subluxations on] your x-rays by hand? Most clinicians are! PostureCo offers the latest and most accurate technology for clinical x-ray digitization’ [123]. ‘PostureRay was released in January 1, 2007. Since this program is already in high demand, you may wish to secure your copy by emailing [address] or calling [phone number]. Only 30 installations per month will be supported so that we can provide the first rate personalized technical support in this first release. Reserve your copy today’ [123].

### Cowin upper cervical orthogonal

- ‘We take X-rays in three planes to see how far the spinal bones have moved from their normal positions. Because the cumulative effects of several upper-neck misalignments, each perhaps as small as 0.75 degrees (or, translated into linear measurements, each as small as 28 thousandths of an inch or 0.7 mm)’ [82].
- ‘The wire artefacts we place on the skin show up on the X-rays to tell us how far the spinal bones are from the ear and other 'outside' structures, allowing us to map both the locations of the spinal bones and the line of access for the spinal adjustments’ [82].
- ‘We fuss about positioning patients for X-ray, and about ensuring they keep their eyes closed, because we want the films to show posture the patient has unconsciously adopted to compensate for the spinal malfunction’ [82].
- ‘Before a patient receives a first adjustment (treatment), we spend an hour or more analysing the X-rays’ [82].
- ‘The analysis is completed when the chiropractor has decided which of about 36 adjustment categories is most strongly indicated by the initial set of films’ [82].
- ‘After the first “trial-adjustment”, we take two new X-rays and compare them with two of the three initial X-rays to find out what changes in vertebral position may have been achieved by this adjustment attempt. Getting the best adjustment, may require several 'trial-adjustments' over a number of visits’ [82].
- ‘The other comparison X-ray in the series (sagittal plane natural lateral) is taken about 18 months later in the hope of finding evidence of improved posture. The chiropractors interpret this kind of improvement as resulting from the patient's new self-help activities over this time’ [82].

### Duff method of analysis

- Upper cervical x-rays are taken on patients [77].

### Gonstead

- ‘…full-spine radiographs are taken in the standing, weight-bearing position to fully substantiate the examination findings’ [57].
- ‘This is helpful in evaluating posture, joint and disc integrity, vertebral misalignments and ruling out any pathologies…’ [57].

### Grostic

- ‘The procedure employs a method of X-ray analysis that quantifies the lateral and rotational misalignments between atlas and axis as well as atlas and occiput. The analytical procedure examines the spatial orientation of the atlas, the geometry of the articulating surfaces, and the misalignment configuration to arrive at an effective correction vector. In addition to the X-ray analysis, the system contains steps for ensuring the precision of the X-ray analysis, adjusting procedures, and post-adjustment re-evaluation procedures’ [149].
- ‘The x-ray analysis is the real core of the procedure and is the one area that has remained constant over the last 30 years’ [149].
- Seven of twenty-one chapters of the official textbook for this technique pertain to x-ray [66]
- ‘Post X-ray assessment was recommended to ascertain that at least 50% correction was achieved after the initial adjustment. Post X-ray assessment is also important to determine if an errant adjustment occurs; and provides information for the doctor to make the appropriate correction(s) for future adjustments’ [149].
- Grostic encouraged the chiropractors attending his seminars to arrive early, be radiographed, adjusted personally by him, then re-radiographed, so they could see the change themselves [66] p.24.
- Kale
- ‘Three x-ray views are required to determine the subluxation of the Atlas (top cervical vertebra); they are AP open mouth, lateral (through C1), and base posterior’ [125].

### Knee chest upper cervical specific (KCUCS)

- ‘6 week intensive course designed to develop you into an expert in Upper Cervical X-ray analysis’ [181].
- ‘Curvilinear Misalignment of C1 in Subluxation. The foundational basis of this concept is rooted in the work of William Blair, D.C., originator of the Blair technique’ [182].

### Logan basic

- Full spine radiography is required [183].
- Ten patterns of ‘distortion’ or what they considered abnormal spinal curvatures, which they marked on the radiographs [49] p.176.

### Mears

- ‘a new method of X-ray analysis … which established more reliable points of reference to determine cervical distortion more accurately. This in turn led to the need to modify the adjusting procedure used in the H.I.O. technique. The new X-ray analysis indicated that segment rotation was a major factor in cervical distortion and it became apparent that a method of adjustment had to be devised which would correct rotational as well as lateral displacement. What developed was a new method of adjustment which with the X-ray analysis became the basis for the Mears Technique’ [152].

### National upper cervical chiropractic association (NUCCA)

- ‘Our profession offers a plethora of organizations to belong to and have membership in. Each has their own agenda and to the extent that agenda resonates with how you practice and what you believe in, is the extent that you would support them. No organization will be perfectly aligned with what you know to be true, but our group represents the doctor who makes NUCCA protocol the mainstay of his/her practice. Just think if you suddenly lost your ability to take post x-rays? There is movement to enforce this change. One of the strongest voices and your most consistent ally to not let this happen is the NUCCA organization. We are aware and watching these developments and the UCRF [Upper Cervical Research Foundation] has tooled its focus to publish research to support the need for post x-rays’ [184].
- ‘Research shows that problems in the cervical spine can cause progressively degenerative structural, neurological, physiological and pathological symptoms. Yet, when a small but measurable spinal misalignment known as the Atlas Subluxation Complex (ASC) is detected and corrected, a return toward good health and normal activity can be realized’ [76].
- ‘We are biomechanically based and depend on objective measurements utilizing pre-adjustment and post-adjustment X-rays along with an instrument called the Anatometer for the measurement of body posture and skeletal distortion. It is our long-term clinical experience that postural distortions compromise the functioning of the nervous system and eventually the overall physiology of the human body’ [172].
- The Atlas Subluxation Complex and Atlas Subluxation Complex Syndrome are ‘…progressive and degenerative, are a detriment to human health and well being and pose a burden on our society and the health care providers treating the various symptoms and conditions associated with them’ [172].

### Orthospinology

- Uses Grostic x-ray analysis [185] including post x-rays [149].
- Outcome measures are radiographic and postural analysis [186].

### Palmer upper cervical specific/HIO

- Radiographs are necessary to demonstrate subluxation [1].
- Post-treatment radiographs show correction [43].

### Pettibon

- ‘The Pettibon Rehabilitation System starts with an x-ray examination. Seven views of the spine are routinely taken, more if necessary’ [100].
- ‘Pettibon x-ray analysis may involve over 41 lines and 23 angles to specifically locate the displaced vertebra’ [101] p.7.
- ‘The initial set of examination x-rays are compared to x-rays taken during treatment to assess progress and ultimately to prove the treatment's success. Whether the x-rays are for diagnosis, testing, assessing progress, post-treatment evaluation and/or proof, patients are seated and the x-rays are always taken, marked, and measured the same way, every time’ [100].
- ‘The initial, interim and post x-rays are specifically analyzed and measured…’ [101] p.33.
- ‘Prospective patients aren’t automatically accepted for care. Doctors need to determine “if” and “how” prospective patients will respond to care. And this requires testing. In one test, prospective patients will wear Pettibon System Weights and have their lateral cervical spine re-x-rayed. Other tests evaluate whether prospective patients’ postural muscles are strong enough for the corrective procedures. Typically, as high as 40% lack the strength and endurance they need. So they will be required to go through a rehabilitation program to strengthen their postural muscles before going on to spinal correction’ [100].
- ‘At the end of acute care, patients are re-x-rayed. The x-rays helps the doctor assess patients’ progress and qualification for the next phase of care. Re-x-raying serves another purpose. If patients have been in an accident, it's only after their muscles are no longer in spasm, guarding the spine, that the exact damage can be seen’ [187].
- ‘Chiropractors claim to "adjust" or "manipulate" the spine. If this is true, the results or changes should be visible on x-rays taken after the adjustment or manipulation. The Pettibon Procedures are one of the few techniques in Chiropractic that cause measurable changes…’ [101] p.7.

### Pierce results

- Pierce was confounded by inconsistent results and so developed a model of a ‘normal spine’ and ‘true subluxation’ and so developed a more rigid system to produce consistent results [188].

### Spinal orthopaedic neurological advancement and research (SONAR)

- ‘SONAR was developed by Thomas Elliott, Jr., D.C. who was a NUCCA practitioner. SONAR employs procedures for taking and analyzing x-rays’ [66].
- ‘…linear and torque forces in the exact angle and amplitude as determined by x-ray analysis’ [152].

### Spinal stressology

- ‘Standing and seated front-view and side-view full spine x-rays are recommended in severe pain patterns, work related injuries, automobile accidents and degenerative disease conditions. A system analysis consists of standing and seated front-view and side-view full spine x-rays. We can scientifically measure abnormal stress, and predict what specific damage it will cause if not corrected’ [189].
- ‘First of all, we sit down and tell them what we need to find on x-rays. If we find there is a difference between standing and sitting patterns, and there is a prognosis, then we will accept them. We also say if the spine is degenerative in both standing and sitting positions, that we cannot accept them, or the prognosis is, in fact, poor. Some of our kids, you know, are totally confined to a wheelchair, and it is very difficult to get standing x-rays on them’ [128].
- ‘A mental-emotional profile is an evaluation based on standing and seated, front and side view, full spine X-rays’ [157].
- ‘Each [radiographic] measurement allows us to help people understand which emotions are theirs and which emotions are inherited from a previous generation’ [157].
- ‘People ask if we have to take full spinal X-rays on every patient to gain awareness of their underlying mental-emotional stress issues? The answer is that pain in the body and short leg deficiency will reveal specific information about what specific stressors are taking place within a person’s life’ [157].
- ‘We are saying that we make no cures and can give no guarantees…’ [128].
- ‘…on every patient we record 40 different measurements of critical landmarks of the spine and their variations as to the systemic normals. So each measurement is classified as to whether it is a breakdown component or whether it is in defense. Then the computer makes its analysis and each measurement is then classified as to whether it is mild, moderate, severe, degenerative acute, degenerative severe, or degenerative out of control. We look at this as a way of measuring subluxations on a systemic basis’ [128].
- ‘We have to evaluate what we've done in order to substantiate what we are doing, and the chiropractic profession doesn't believe in re-evaluation and they haven't put together a subluxation system that would warrant post x-ray analysis. I think this is too bad and is really a short vision type thing’ [128].

### Sutter specific atlas correction

- Radiographic interpretation is a combination of standard HIO and elements that Sutter invented himself [177].

### Zimmerman

- ‘Two views taken down through the hole-in-the-head [sic] plus a side view and a front to back view constitute an X-Ray [sic] set. Analysis of these X-Rays provides the information needed to make the proper adjustment’ [190].

## Appendix 4: sacred stories

### Advanced orthogonal

Case reports, patient testimonials, practitioner testimonials, and unsupported extrapolations from basic anatomy: ‘…in simple terms the human skeleton is “top down design” not “bottom/feet up”. This means your posture is driven by the position of your head in space’ [160]. Another type of story is related in the concept of ‘holding a correction’ as determined by radiographic analysis [159].

### Applied spinal biomechanical engineering

Practitioner testimonials and patient testimonials, [191] and vague definitions for the practice methods with difficult to decipher language: ‘ASBE identifies and reduces spinal distortion through the rehabilitation of normal muscle tissue, restores the normal physiologic dynamics of the spine, and treats within the natural parameters of the individual patient (bilateral symmetrical function is secondary to static non-dynamic gravitational dependent equilibrium)’ [83] p. 2. ‘Most patients are amazed once they see their X-rays up close’ [192].

### Applied upper-cervical biomechanics

Essays about the existence of subluxation and its ‘correction’ proven by the use of thermal (infrared) imaging, as well as case studies that focus on claimed benefits to patients with multiple organic syndromes like cortical blindness, epilepsy, autism, asthma, etc. [193].

### Atlas orthogonality

Patient [105] and practitioner testimonials [194]. One case series is cited as well as several case reports. Unsupported extrapolations from basic anatomy with oversimplified analogies are also used: ‘The nervous system can be likened to a major super highway. Without problems and all vehicles going the speed limit, travel is smooth and without accident. However, if there is a car crash, traffic slows down and even stops. The highway does not function as it was intended’ [144]. ‘This one bone can effect [sic] the alignment of the entire spine. The spine is like a chain—when the first link is twisted and turned, each link down to the last turns—thereby disrupting the rest of the chain. Consider the atlas the first and therefore the most important link in that chain’ [144].

### Blair

[Chiropractic] helped Blair’s chronic bronchial asthma so much that he decided to become a chiropractor [66] p.30.

Extrapolations from basic science: ‘All nerve messages between the brain and body must pass through the upper cervical area’ [69]. Appeal to authority epistemology: ‘Dr. Palmer conducted studies in Germany on cadavers and found that the brain stem or medulla, extended into the neural canal down to the level of the lamina of the second cervical vertebrae, at which point it becomes the spinal cord extending downward. The brain stem has been referred to as "Houston Control". It is the area where nerve cell centers are located that control many of the major functions of the body…’ [98]. ‘If for example the atlas is impinged against the part of the cord that sends messages to the left hand, that individual may experience a numbness, burning or tingling sensation in that hand’ [98]. Note: These symptoms are lower motor neuron signs and would be present if a peripheral nerve was impinged, not if the spinal cord or medulla oblongata were. ‘If the nerve tracts at the brain stem level go to the heart are being impinged that individual may experience high blood pressure, palpitations or an irregular heartbeat’ [98]. ‘Any part of the body can be effected [sic] when there is pressure on the brain stem or spinal cord because almost all of the nerves have to pass through this area before reaching the part of the body they innervate’. ‘WHEN [sic] we turn the fan off it does not stop immediately because the momentum must ease down and stop. When we turn the fan on it does not reach its maximum r.p.m.'s [sic] immediately because it must pick up or gain momentum. I, [sic] think you appreciate our idea of momentum, and resistance. In applying this to your body, recognize that your body is living under a condition of momentum. Every impression you receive is more or less resisted by the tissue cell. Let's consider a physical force such as a fall. The invasion of force to the body caused by the fall overcomes the resistance of the body internally. These momenta coming together causes a clash of forces. If the invasive force is greater than the internal resistive force, the shock waves or forces cause the vertebral subluxation. Now that the vertebral subluxation has been produced an alteration or interference of life energy (nerve impulses) causes a dis-eased condition to develop in one tissue cell, two cells, then 200, 2,000 and then 2 million. Proportionately, the longer the subluxation exists, the greater becomes the momentum of speed of diseases. The longer it runs, the longer you go down hill, the greater becomes it's [sic] speed and the greater becomes the momentum of disease’ [111]. ‘A subluxation may be present for months or years before producing any outward signs such as pain or symptoms, causing the body to break down to a state of diseases’ [98]. ‘The purpose of the Blair Chiropractic technique is not to diagnose or treat diseases or conditions, but to analyze and correct vertebral subluxations in an accurate, precise and specific manner to allow the body's intelligence, (see chiropractic philosophy) to mend, repair and maintain health from within’ [98].

### Chiropractic BioPhysics

In addition to case reports, CBP utilised case anecdotes: ‘…studies have shown that many CBP patients have also seen a dramatic improvement of other health issues, including ADD/ADHD, Asthma, Blood Pressure, Chest Pain, Chronic Fatigue, Constipation, Fibromyalgia, Headaches, Hypertension, etc.’ [167].

Distorted evidence: ‘In CBP® Technique, the use of initial and follow-up spinal x-rays or radiographs is deemed necessary; however, some in chiropractic have condemned the use of follow-radiographs to collect alignment data. Importantly, there is data [sic] to show that the use of medical/chiropractic x-rays constitutes a very minor health risk and in fact has been shown to be of benefit (decreased sickness and cancer mortality rates) in some studies’ [114].

Oversimplified analogies: ‘In reality, the only way to see what an individual patient’s spine alignment looks likes, is to obtain spinal imaging such as Radiography or X-ray. No-one would not [sic] take their car to the mechanic and say: Something’s wrong with my engine but don’t look under the hood – Would you? Then why would anyone want a Chiropractor to adjust-treat their spine [sic] without having an x-ray to see what the person’s spine looked like? Would You?’ [114].

### Cowin upper cervical orthogonal

Case anecdotes [195] and a single case report [196]. ‘A surprising number of stubborn health problems have been helped by chiropractic care over the past 106 years. Current conjectures for the successes reported in the past century include the following component ideas. Most persistent health problems have more than one cause’ [68]. ‘A frequent contributing cause is somatic disturbance, such as loading distortion on the supporting structures, causing the highly movable spinal bones to lose their proper alignment’ [68]. ‘The somatic disturbance most frequently assessed and treated by chiropractors, called a subluxation, is a persistent and/or recurring, small alteration in alignment, movement integrity and/or physiological function in one or more spinal motion segments’ [68].

### Duff method of analysis

Patient testimonials [106] and a mission statement which reads: ‘Health and Happiness are Synonymous’ [121].

### Gonstead

Clarence Gonstead entered the profession on the belief of his own personal rheumatoid arthritis cure at the hands of a chiropractor. The other story about Gonstead personally was that he was somehow superhuman, with claims that while in practice he adjusted 250 patients every day in the 1940s, and even more in subsequent decades, increasing his office hours and often finishing the last patient at 2:30 am [75].

Practitioner testimonials [197] and case reports [198]. Case reports were solicited on the website: ‘We are looking for both cases which you successfully helped and those you didn’t and might have even referred out [sic]. We are also looking for cases that you might consider ordinary or routine, as these are rarely presented, but we often see patients with these presentations. This is the minimum that we would like if you want to submit a case study for the newsletter and web site. We have removed some parts that are required for peer-reviewed journals and have emphasized some to provide a Gonstead perspective. If you absolutely cannot write, send us whatever you have, and we will work with it’ [147].

Oversimplified analogies: ‘The Gonstead Concept of chiropractic begins with a basic biomechanical principle of physics. Every engineer, architect, builder and carpenter knows the importance of a proper foundation in constructing a building, for this insures durability and long life. Any slight change or shift in the foundation can cause a great amount of deviation in the top part of the structure and, perhaps, ultimately, its collapse. When the pelvic girdle or any of the vertebrae (bones making up the spinal column) become tilted or rotated out of their proper position, dramatic changes may occur in the body’ [120].

Assertion of superiority over upper cervical systems: ‘If just the top misaligned vertebra was adjusted, in cases where additional vertebrae are misaligned, only limited relief could result. This would not be getting to the source of the trouble. For complete and lasting results, all of the misaligned vertebrae must be identified and then a program can be initiated to restore them to their normal position’ [120].

### Grostic

Sacred stories included the above-mentioned tales of Grostic’s health and restoration, his superhuman practice schedule, case reports, case anecdotes, and theories on the mechanism of vital energy flow and health [66]. Several paragraphs of the official textbook were a treatment of the concept of spinal irritation; they quoted texts from the 19th century and their discussions of how bones may impinge the flow of nervous energy, leading to derangement of the functions of organs [66] p.2. This information is presented as relevant and is reprinted without questions or clarification.

‘The evidence that ‘specific’ upper cervical chiropractic is effective in promoting wellness is compelling and widespread. You need only look for it’ [149].

‘…the peer-reviewed literature… shows a documented correlation between orthogonally-based care (Grostic/ Orthospinology, NUCCA and Atlas Orthogonality) and the improvement of various patient complaints’ [149]. There followed a list of 22 papers claiming resolution of cases of various conditions, including seizure disorders, Tourette’s syndrome, and congenital brain malformations, one of which appeared in a peer-reviewed journal that is included in PubMed [149]. The anecdotes continued with broad claims: ‘Athletes and people looking to achieve peak potential performance also seek Chiropractic care to have the best possible brain body connection so their reaction time and endurance is at it's best. Many pregnant mothers have experienced the benefits of shorter births, easier labor, and pain free pregnancies while under Chiropractic care’ [199]. The stories also included extrapolation of anatomical findings. ‘minute amounts of atlas [upper cervical vertebra] laterality or rotation were important from a neurological standpoint…’ [66] p.19. ‘The two most plausible hypotheses have to do with spinal cord tension and mechanoreceptive dysafferentation’ [149].

### Kale

‘By eliminating nerve interference, Specific Chiropractic, ("Brain Stem Specialist") can correct numerous health problems and help prevent many conditions from developing. This enables the body to maintain its natural, inborn resistance to illness and disease. For you to have a longer, healthier and happier life, it is imperative that you be aware of the fact Subluxation of the top two vertebrae is a serious condition and can easily develop conditions affecting every part of the body. It has been estimated that as much as 90% of the world's population suffers from this type of Subluxation in varying degrees interfering with the normal function of the body, and therefore, interfering with and preventing health. Don't wait for health threatening, or even life threatening conditions to develop. Have a "Brain Stem Specialist" evaluation today’ [200].

‘The spinal column is intended to protect the delicate Brain Stem [sic] and the nerves leading from it. When it becomes subluxated, the column does not protect. Instead, the misaligned spinal bones of C1 and C2 impinge the Brain Stem. This causes interference with normal nerve function to all parts of the body. When there is interference in normal nerve function, there will be health problems, sickness and disease’ [200].

‘For you to have a longer, healthier and happier life, it is imperative that you be aware of the fact Subluxation of the top two vertebrae is a serious condition and can easily develop conditions affecting every part of the body. It has been estimated that as much as 90% of the world's population suffers from this type of Subluxation in varying degrees interfering with the normal function of the body, and therefore, interfering with and preventing health. Don't wait for health threatening, or even life threatening conditions to develop. Have a Specific Upper Cervical Chiropractic evaluation today’ [201].

‘Subluxation of the top two vertebrae, C1 and C2, Atlas-Axis, close down the opening of the neural canal in this area and result in pressure upon the "Brain Stem", thus causing impingement of spinal nerve tracts. This can result in malfunction in all parts of the human body with resulting pathological changes. Mild to moderate malfunction results in sickness and disease, while extreme malfunction results in DEATH!!’ [97]. [emphasis original] ‘The patients… had positive results and some had complete recovery from such problems as Parkinson's Disease, AIDS, Paralysis, Sight and Hearing disorders [74].

### Knee chest upper cervical specific (KCUCS)

‘Upper Cervical Care is not new to healthcare; it was first introduced back in the early 1920’s. Dr. BJ Palmer began his endeavor back in 1910 when x-ray was introduced and visualization of the spine was made possible. In 1935 the Research Clinic was opened and began seeing patients. From 1935–1942 5,000 cases were seen and of those, 3,856 made a full recovery, 1,013 cases reported improvement, only 49 or 0.9% reported no improvement, with 82 or 1.6% unreported. 97.3% were recovered or improved. Then in 1942 – 1954 they began to only except cases that had no prior success with medicine or otherwise. 139,096 cases were seen, 63.8% (88,743) made a full recovery, 29.4% (40,894) improved, and 6.7% (9,319) no change’. There were also four case reports supporting the KCUCS system, one of which was published in a peer-reviewed journal that is included in PubMed [202] as well as case anecdotes [171].

### Logan basic

‘We can prove conclusively that the sacrum and coccyx are the primary portion of the primary curvature of the spinal column-“as the sacrum goes so goes the spine”-and that it is absolutely necessary to restore the sacrum to normal position and relationship with articulating bones to effectively reduce curvatures, subluxations, and disease’ [151].

### National upper cervical chiropractic association (NUCCA)

Case anecdotes [203,184] and extrapolations from basic anatomical observation [122] were both present.

‘It is the experience of upper cervical chiropractors (2-3% of all chiropractors) in general and NUCCA practitioners in particular that there is no anatomical region of the human body that is so commonly a first cause of postural problems as the upper cervical spine. Specifically this first cause is itself often the effect of injury from trauma, surgery, applied mechanical forces, or even age itself. This site of injury can be at any location; either above, at, or below C-1. The C-1 joint, composed of C0-C1-C2, is the focus of upper cervical work. It is well known that at no other location in the spine are the joint mechanoreceptors as dense, motion so allowed, and structures below and probably above so dependent’ [122]. ‘The design and development of the Anatometer by Dr. Gregory and Peter Benesh, an instrument which measures bodily distortions before and after the C-1 adjustment, providing proof of the effects of a C-1 subluxation’ [102].

The Mission Statement includes: ‘Maximizing the human health potential associated with the reduction of the Atlas Subluxation Complex (ASC)’ and ‘Maintaining an effective, passionate, responsible organization contributing to the standards of the measurable, objective and reproducible reduction of the Atlas Subluxation Complex’ [204].

‘The Atlas Subluxation Complex includes the measurable misalignment of the head, atlas and cervical spine, the contracted spinal musculature, postural distortion and short leg phenomena. The Atlas Subluxation Complex Syndrome includes the ASC and its effects on the central and peripheral nervous system and the relationship to the symptoms and conditions directly related and those associated with this progressive degenerative condition’ [76]. Misalignments of less than a degree can cause systemic disorders: ‘…there is only a very small range of neurological alignments possible…’ for example, the atlas must be laterally displaced ‘…less than 0.75 degrees…’ [205,122]. ‘…UCRF (Upper Cervical Research Foundation) research provides the validation to understand the wide variety of negative effects that a misaligned upper cervical spine has on human well-being, and provides answers on how to best correct the misaligned spine, to determine the practice limits of the upper cervical specialist, to determine the extent that a misaligned upper cervical spine affects the entire human organism, and to establish the necessity for insurance coverage and other support by involved agencies’ [206].

### Orthospinology

Patient testimonials [207]. ‘Spinal misalignments cause abnormal input to the nervous system. This phenomenon is referred to as the Vertebral Subluxation Complex’ [186]. ‘Abnormal input to the nervous system creates Body Imbalance, resulting in postural distortions that manifest as head, neck and back pain, numbness, tingling, and pain radiating into the arms and legs, and compromised general health’. ‘Chiropractic is the science of detecting and correcting spinal misalignments that cause irregular input to the nervous system, restoring balance to the body and its systems. Normal health is the natural result’ [186]. ‘Because the upper cervical spine connects to the brainstem, a misalignment there may also disrupt brain to body communication leading to symptoms such as headaches and migraines, sinus problems, vertigo and blackouts, seizures, TMJ and facial pain as well as fatigue, immune compromise and general health deterioration. Even before symptoms manifest, this misalignment called the Vertebral Subluxation Complex, may be limiting your bodies’ ability to function at the highest level creating an internal environment conducive to the development of disease’ [186].

Oversimplified analogies: ‘The nervous system works like a telephone network carrying messages between the brain and the body. When the nervous system is working correctly, the body has the proper information to heal itself and maintain balance of all your systems. Static on the telephone line interferes with the messages being sent. If the nervous system has "static" (irregular input), the messages between the brain and the body will be interrupted. A misalignment of the vertebra of the spine may cause distortion, and body imbalance results. When a Vertebral Misalignment causes the nervous system to initiate imbalances of the body systems, it is known as a Vertebral Subluxation Complex, or in simpler terms: loss of body balance. Upper Cervical doctors are trained to determine exactly how the upper cervical spine has misaligned in your neck and the set-up and exact angle necessary to correct it. Once the Vertebral Subluxation Complex is corrected and the “static” removed, the body can return to a state of balance’ [186]. A video is provided on the official website, apparently showing an unconscious patient with a known seizure disorder revived by application of this technique [173].

### Palmer upper cervical specific/HIO

The writings of BJ Palmer are replete with case anecdotes, [43] as is commonly known.

### Pettibon

Case anecdotes [208] as well as patient and practitioner testimonials [107]. ‘Our purpose: To energize lives through spine & posture rehabilitation’ [209]. Pettibon and Vern Pierce ‘…discovered how chiropractic students—about 100 from Palmer and Life—who entered college with x-rays showing normal or near normal lordotic cervical curves, on comparison x-rays taken at the end of their schooling, had abnormal military or kyphotic necks, and most had spinal pathology’ [113]. Sharon Freese-Pettibon ‘…did what Dr. Pettibon prescribed and two weeks later was eating solid food for the first time in three months, her bleeding ulcer was starting to heal and she was beginning to feel better.’ After a surfing accident, ‘In three visits the paralysis was totally gone and she was able to return to Hawaii with her health intact’ [113].

Extrapolation from basic science: ‘Gravity is an absolute environment to which the upright spine and posture of humans must develop and relate. Since gravity is an absolute, there has to be an absolute optimal position for the upright spine and posture. The skull is a vertebra. It's the only vertebra that knows its neurologically optimal position and has the ability to establish and maintain that posture. The global spine's position relative to gravity is more important than its units or its segments. A less than optimum lateral and A-P spine and posture compromises spinal function’ [210].

Distorted evidence: ‘Debunking the Myth About X-Rays with Facts: The accepted cumulative dose of ionizing radiation during pregnancy is 5 rads The most sensitive time during pregnancy is between 10 and 17 weeks. Two routine chest x-rays = .00007 rad. Radiation to the fetus shouldn't exceed 10,000 millirads. Annual environmental radiation is 300 millirads/year which equals 12 chest x-rays. The Pettibon X-ray Series (7 views) = 20 millirads. To exceed 10,000 millirads would take 3,124 x-rays’ [100]. No references were cited for this information.

### Pierce results

‘… high quality chiropractic care can not only save the lives of your patients, but your practice as well as our profession!’ [188]. 6 case anecdotes, including ‘100 percent recovery!!!’ from headaches, low back pain, heart palpitations, migraines, and degenerative disc disease (‘Osteophytic bridge being RESORBED!!!’ ‘Notice IVD regeneration’) [211].

### Spinal orthopaedic neurological advancement and research (SONAR)

‘Not having your atlas corrected to restore body balance is like continuing to drive a car that is out of alignment. Over time, everything is going to break down. You can keep replacing the tires, but unless you have the car aligned, you'll just be wasting your money. Similarly you can keep taking medication, but if your atlas is out of its proper position, causing the problem, you're not going to get well until you get your atlas corrected’ [155]. ‘…if the atlas has moved out of position even a fraction of a degree very serious things can result’ [212]. ‘The power that made the body heals the body, it happens no other way’ [213].

### Spinal stressology

Patient testimonials [214] and case anecdotes [214]. Muscular dystrophy ‘…is a psychosomatically-induced disorder…We find that usually it is referenced to inherited abortion, inherited abandonment, and inherited adoption processes where something of this nature has occurred in parents, grandparents, or even great grandparents, and the hostility of that is passed on, and we believe that muscular dystrophy becomes a psychosomatically-induced illness wherein the child unconsciously develops a covert disease process that will lead to his death’ [128].

‘I just lost a son-in-law, 35 years old, whose x-rays showed, back in 1986, that he inherited death wishes… I should not say the x-rays showed; the personality analysis of the psychological coefficients from the x-ray findings would tend to indicate that he had multiple death wishes’ [128].

‘Well, it's kind of interesting that in the article on "SIDS Death", they found that the average atlas angle was at 30 degrees. Now, when we find 30 standing and 29 sitting, the 29 indicates an intent to commit suicide and the 30 indicates that the person tried to commit suicide, so we feels that even in SIDS death that there is a fetal suicide’ [128].

‘Comparative radiographs are justified and mandatory for monitoring patient progress and for evaluating treatment effectiveness. -From the comparative x-rays an updated diagnosis and prognosis is ascertained. Documentation establishes objectivity’ [215]. ‘When you see an X-ray of the entire spine, you start to understand the why’ [215]. ‘Dr. Ward has found that disease, both physical and mental, shows up in a specific spinal curvature pattern’ [176]. ‘Maybe we shouldn’t be surprised to discover that your spine reflects your personality, or that today’s stress was passed down from a previous generation’ [216]. ‘Each measurement allows us to help people understand which emotions are theirs and which emotions are inherited from a previous generation’ [216]. ‘When a person is driven in their behavior, the spine always goes forward to normal. The neck goes forward, the mid-back curve goes forward, the lumbar curve reverses. People who are most driven are forward in both the standing and sitting position. A forward spinal curve relates to the driven mother, grandmother and or great grandmother. When the spine begins to go forward, the person will begin to feel exhausted. The more the spine deviates forward the more immune compromised the person will be’ [216].

### Sutter specific atlas correction

Rejection of scientific enquiry: ‘Any attempt to explain how these elements have come to be arranged into this complex, self-regulating "machine", how these elements have come to feel, think, and live as one unified whole, upon a purely material basis, is doomed to failure in advance’ [129].

‘You will note that our examination is largely confined to the spinal column, and particularly to the topmost part of the spinal column immediately below the head. This is because our research work has proved that practically all of the disturbances of nerve structures which interfere with control of normal body function take place in the region of the first spinal bone or vertebra called the atlas. Our object in making this examination is not to diagnose disease as does the physician, but to gain information regarding the cause of the interference that is preventing Innate Intelligence from curing. When this cause is corrected Innate Intelligence immediately starts the curing process. The disease may be in the stomach, the liver, the heart, the intestines, or any other part of the body, but wherever it is Innate Intelligence knows, and is the only one who knows, exactly what to do about it. The best doctor in existence is the Intelligence that God left in charge of our bodies…’ [129].

‘The effects of such nerve interference may not show up for some period of time. It is much easier and better to correct the cause of such interferences as they occur and thus prevent the development of some condition than to correct a condition once well established. Should anything unusual in the way of a fall or sudden strain or jar to the head or neck occur, it would be advisable to have the atlas checked as soon as possible. Once a month would be a good average interval for checking the condition of the atlas vertebra under usual circumstances. Develop the once a month check-up habit. It is inexpensive health insurance’ [129]. ‘Only when symptoms disappear, following the correction of the cause of the condition within the body, as a result of regeneration by the Innate Intelligence within the body has anything really worth while and of lasting benefit been accomplished [129].

### Zimmerman

Patient and practitioner testimonials [190]. ‘A chiropractor who is skilled in the art of adjusting by machine can free the spinal cord from pressure and get the patient well’ [56]. ‘The basic natural premise behind the idea is the fact that life depends on a connection from the brain to the body through the central nervous system. This connection is in the form of nerve fibres leading down the spinal cord from the brain to all parts of the body. These fibres (there are about 10 billion of them) are like wires in a telephone cable. They are subject to pressure at the base of the skull when the atlas or axis vertebra is out of its normal alignment’ [56]. ‘When pressure is removed from the spinal cord, the effect is the same as turning on the electric power to a light bulb. Without the electricity the lamp does not produce any light. When the patient's body is deprived of energy by a nerve pressure, anything can go wrong and death may occur. All it takes to restore the flow of energy to the sick body is ONE accurate adjustment’ [56]. ‘Trauma is the origin of most subluxations, even back to “birth trauma” including forceps delivery’ [56].

## Appendix 5: special language

### Advanced orthogonal

With this technique, special language includes the term ‘orthogonal’ meaning ‘in normal position’. The standard definition of orthogonal is ‘at right angles’. In addition, the x-ray views, ‘nasium’ and ‘vertex’ are not standard views taught to or utilised by medical radiographers and so the names can be considered special language. The term ‘three dimensional x-rays’ is also specially defined here, meaning x-rays taken from three different angles, not the creation of a 3-D image as the term is understood for advanced imaging or modern movies and television.

### Applied upper-cervical biomechanics

The non-standard radiographic views of nasium and ‘base posterior’ [162] as well as ‘arthrokinematic radiographic analysis’, and ‘neurophysiological imaging’ [143].

### Atlas orthogonality

‘Orthogonal’ as used by the technique, as was seen with Advanced Orthogonal technique (above). The term ‘three dimensional x-rays’ was also the same as that used by Advanced Orthogonal and has the same meaning, that is, x-rays taken from three different angles, not the creation of a 3-D image.

### Blair

The names of some of the radiographic views, ‘Blair Oblique Protractoviews’, ‘base-posterior’ and the terms for some of the equipment for radiographic imaging and analysis: ‘D-arm protractor’ and ‘Blair Protracto clamp’, used in radiography. Another term, undefined, was used in this way: ‘The presence of cervical nerve interference is established by observation of both persistent differential paraspinal derinothermographic pattern and functional leg length deficiency’ [103]. Although ‘derinothermographic’ may or may not have been directly related to radiography, it is included here because of its stated close relation to x-ray analysis of the subluxation.

### Chiropractic BioPhysics

‘PostureRay’ software for x-ray subluxation analysis as well as the name of the technique and its acronym, and the names for non-mainstream radiographic views like ‘nasium’. The special language of this technique claimed propriety for its name and devices, like PostureRay, which were rarely found without the® registration symbol.

### Cowin upper cervical orthogonal

Non-standard x-ray view names (e.g. nasium, vertex, etc.) [82].

### Gonstead

Highly detailed vocabulary of subluxation ‘listings’ [124] or descriptions of perceived spinal misalignments, and was bolstered recently to help bring all practitioners of the system into line. ‘The GCSS Gonstead Technique Terminology Project began in response to a highly inaccurate 2002 article on the Gonstead System…’ [217].

The terms ‘split’ or ‘gradient’ screens: ‘In the past, and not uncommonly today, x-ray screens of different sensitivity speeds were used to compensate for the different body densities’ [124].

### Grostic

The non-standard x-ray view names (e.g. nasium, vertex, etc.), ‘cephalocentroscope’.

### Kale

The non-standard x-ray view names (e.g. nasium, vertex, etc.).

### Knee chest upper cervical specific (KCUCS)

Similar to that used by Blair, as well as ‘Spiz’.

### Logan basic

X-ray marking system [218].

### National upper cervical chiropractic association (NUCCA)

The non-standard x-ray view names (e.g. nasium, vertex, etc.), and ‘anatometer’ [219].

### Orthospinology

The non-standard x-ray view names (e.g. nasium, vertex, etc.), [185] and spiz or spizzerinctum [220].

### Pettibon

Non-standard x-ray view names (e.g. base-posterior), and postural change descriptors (e.g. cervical dorsal angle, upper angle, lower angle, etc.) [221].

### Pierce results

Subluxation. Atlas ‘out of compensation’ [211]. ‘Spinal visualizer’ believed to mean cineradiography [211]. ‘Master Level’ certification, [222] not through a university.

### Sutter specific atlas correction

None found other than ‘subluxation’ and ‘nerve interference’ [129].

### Zimmerman

None found other than ‘subluxation’ [56].

## Additional files

Additional file 1:
**List of named techniques that used plain radiography for subluxation detection.**
Additional file 2:
**Summary of religious characteristics in each technique.**
