# Supplementary material for: Gimme that old time religion: the influence of the healthcare belief system of chiropractic’s early leaders on the development of x-ray imaging in the profession
Source: Chiropr Man Therap. 2014 Oct 28;22:36. doi: 10.1186/s12998-014-0036-5 (PMC4228104; doi:10.1186/s12998-014-0036-5)
Supplement: Additional file 1: — List of named techniques that used plain radiography for subluxation detection. [file 12998_2014_36_MOESM1_ESM.docx]

| **List of named techniques that used plain radiography for subluxation detection** |
| --- |
| Advanced Orthogonal |
| Applied Spinal Biomechanical Engineering (ASBE) |
| Applied Upper Cervical Biomechanics (AUCB) |
| Atlas Orthogonality |
| Blair |
| Chiropractic BioPhysics (CBP) |
| Duff Method of Analysis |
| Gonstead |
| Grostic |
| Kale |
| Knee chest upper cervical specific (KCUCS) |
| Logan Basic |
| Mears |
| National Upper Cervical Chiropractic Association (NUCCA) |
| Society of Chiropractic Orthospinology |
| Palmer Upper Cervical Specific (HIO – Hole in One/Toggle Recoil) |
| Pettibon |
| Pierce/Stillwagon |
| Spinal Orthopedic Neurological Advancement and Research (SONAR) |
| Spinal Stressology |
| Sutter Specific Atlas Correction |
| Upper Cervical Orthogonal (Cowin) |
| Zimmerman |
