# Supplementary material for: Gimme that old time religion: the influence of the healthcare belief system of chiropractic’s early leaders on the development of x-ray imaging in the profession
Source: Chiropr Man Therap. 2014 Oct 28;22:36. doi: 10.1186/s12998-014-0036-5 (PMC4228104; doi:10.1186/s12998-014-0036-5)
Supplement: Additional file 2: — Summary of religious characteristics in each technique. [file 12998_2014_36_MOESM2_ESM.docx]

| **Technique name (originator(s))** | **Supernatural concepts** | **Claims of supremacy (examples)** | **Rules/rituals** | **Sacred artefacts** | **Sacred stories** | **Special language** |
| --- | --- | --- | --- | --- | --- | --- |
| Advanced Orthogonal (G Stanford Pierce Sr and Jr) | Vitalism  Supernatural calling | Most accurate assessment of spinal misalignment  ‘Most evolved’ upper cervical procedure  Practitioners have additional education  Practitioners have higher expectations for correcting patient subluxations  Digital x-ray analysis to measure misalignments to the 1/100th of a degree | Subluxation is primary cause of disease  Upper cervical spine most important  Radiographs must be obtained  The same set of radiographs is obtained on every patient  The radiographs are analysed the same way every time | X-ray machine  Radiographs  Special head clamps for radiographic positioning | Case reports  Patient testimonials  Practitioner testimonials    Unsupported extrapolations from basic anatomy | Subluxation  The term ‘orthogonal’ as defined by the technique system  The concept of ‘holding a correction’  3-D x-ray analysis  Non-standard radiographic views |
| Applied Spinal Biomechanical Engineering - ASBE (Ronald J Aragona) | Vitalism | Doctors discover how little they knew before they started learning these methods and procedures  Provides ‘remarkable advantage’ over other techniques  Dependable and proven  Based on 12 volumes of research | Subluxation is primary cause of disease  Radiographs must be obtained  The same set of radiographs is obtained on every patient  The radiographs are analysed the same way every time  Post treatment radiographs to ‘prove’ correction | X-ray machine  Radiographs | Patient testimonials  Practitioner testimonials  Vague definitions for practice methods with difficult to decipher language  Patient ‘amazement’ at seeing their radiographs. | Subluxation  Difficult to decipher definitions of methods (e.g.‘…bilateral symmetrical function is secondary to static non-dynamic gravitational dependent equilibrium.’) |
| Applied Upper Cervical Biomechanics (AUCB) (International Upper Cervical Chiropractic Association (IUCCA)) | Vitalism | ‘…complex and unique form of radiographic analysis.’  ‘…full body neurophysiologic benefits…objectively substantiated…’  ‘…15 years of unprecedented research…’ | Spinal subluxation is primary cause of disease  Upper cervical spine most important  Radiographs must be obtained  The same set of radiographs is obtained on every patient  The radiographs are analysed the same way every time | X-ray machine  Radiographs  Special head clamps, chair, and laser alignment system for radiographic positioning | Case reports  Essays about subluxation  ‘Correction’ of subluxation ‘proved’ by thermal imaging | Subluxation  ‘neuropathophysiology’  ‘arthrokinematically’  ‘neurophysiological imaging’  Non-standard radiographic views |
| Atlas Orthogonality (Roy Sweat) | Vitalism | Improving the health and extending the lives of people around the world  ‘…the most precise atlas correction…’  ‘There is no guess work in this program.’ | Spinal subluxation is primary cause of disease  Upper cervical spine most important  Radiographs must be obtained  The same set of radiographs is obtained on every patient  The radiographs are analysed the same way every time  Post treatment radiographs to ‘prove’ correction | X-ray machine  Radiographs  Special head clamps for radiographic positioning | One case series  Case reports  Patient testimonials  Practitioner testimonials    Unsupported extrapolations from basic anatomy with oversimplified analogies for lay people | Subluxation  The term ‘orthogonal’ as defined by the technique system  3-D x-ray analysis |
| Blair (William G Blair) | Vitalism  Supernatural calling | This “blue print” of your neck [created from the x-rays] allows the Blair chiropractor to deliver the adjustment that is exactly what your body needs  Allows practitioners to take x-rays and give adjustments ‘with confidence and ease.’ | Spinal subluxation is primary cause of disease  Upper cervical spine most important  Radiographs must be obtained  The same set of radiographs is obtained on every patient  The radiographs are analysed the same way every time | X-ray machine  Radiographs  Rubberised ear markers, Blair ‘protracto clamp’ and special chair for radiographic positioning  Custom viewbox or stereo binoculars to look at the radiographs | Silent killer subluxation  Upper cervical adjustments ‘cured’ Blair’s lifelong asthma.  Unsupported extrapolations from basic anatomy with oversimplified analogies for lay people | Subluxation  The concept of ‘holding the adjustment’  ‘derinothermographic’ pattern  Blair Protracto clamp  D-arm protractor  Non-standard radiographic views |
| Chiropractic BioPhysics (Don and Glenn Harrison) | Vitalism | ‘…higher level of chiropractic.’  ‘…will contribute to building a more stable and successful chiropractic practice.’  ‘…the best technique in chiropractic.’ | Spinal subluxation (postural change) is primary cause of disease  Radiographs must be obtained  The same set of radiographs is obtained on every patient  The radiographs are analysed the same way every time  Post treatment radiographs to ‘prove’ correction | X-ray machine  Radiographs | Case reports  Case anecdotes  Belief in radiation hormesis  Unsupported extrapolations from basic anatomy with oversimplified analogies for lay people  Misleading information about the dangers of ionizing radiation | BioPhysics  PostureRay  Non-standard radiographic views  Several words/names particularly highlighted as special language due to protection with ® symbol |
| Cowin Upper Cervical Orthogonal (Robert Cowin and Kathleen Bras) | Vitalism | Our methods were observed by Wollongong University mathematicians Aldis and Hill and written up in the Journal and Proceedings of the Royal Society of NSW in 1979. | Spinal subluxation is primary cause of disease  Upper cervical spine most important  Radiographs must be obtained  The same set of radiographs is obtained on every patient  The radiographs are analysed the same way every time  Post treatment radiographs to ‘prove’ correction | X-ray machine  Radiographs  Metal artefacts to help reference subluxations | Case anecdotes  A single case report  Patient testimonials  Unsupported extrapolations from basic anatomy | Subluxation  Non-standard radiographic views  ‘adjustic’ |
| Duff Method of Analysis (Stephen A Duff ) | Vitalism  Supernatural calling? | ‘Dr. Duff's speciality is in aligning the upper top two vertebrae…’  ‘…tailor-made adjustment…’ | Spinal subluxation is primary cause of disease  Upper cervical spine most important  Radiographs must be obtained  The same set of radiographs is obtained on every patient  The radiographs are analysed the same way every time | X-ray machine  Radiographs | Patient testimonials  ‘Mission Statement: Health and Happiness are Synonymous. | Subluxation |
| Gonstead (Clarence Gonstead) | Vitalism  Supernatural calling? | One of the most advanced and scientific methods [of correcting] spinal misalignments, joint dysfunctions and subluxation complexes’  ‘…as specific, precise and accurate as possible…’  ‘The Gonstead Chiropractor goes beyond what many chiropractors consider a spinal assessment…’ | Spinal subluxation is primary cause of disease  Radiographs must be obtained  The same set of radiographs is obtained on every patient  The radiographs are analysed the same way every time | X-ray machine  Full spine radiographs  Split screen/gradient screen x-ray cassettes or special s-ray filtration devices | Adjustments ‘cured’ Gonstead of rheumatoid arthritis when he was a child.  Gonstead’s apparently superhuman practice schedule  Practitioner testimonials  Case anecdotes  Unsupported extrapolations from basic anatomy with oversimplified analogies for lay people (fables)  Further reading recommended includes a pro-chiropractic magazine, newspaper, and ‘journal’ | Subluxation  Gonstead system of subluxation description on radiographs.  ‘Split’ or ‘gradient’ screens |
| Grostic (John F Grostic) | Vitalism  Supernatural calling? | ‘…total care of the patient, not simply a spinal adjusting technique.’  ‘…mathematical measurements taken from your x-ray films give the doctor the correct formula to realign your spine.’  ‘…revolutionary advances by Dr John F Grostic…’ | Spinal subluxation is primary cause of disease  Upper cervical spine most important  Radiographs must be obtained  The same set of radiographs is obtained on every patient  The radiographs are analysed the same way every time  Post treatment radiographs to ‘prove’ correction | X-ray machine  Radiographs  Skull measuring device, the cephlocentroscope, used with the radiographs  Head clamp | Grostic’s cure of Hodgkin’s lymphoma, twice, by BJ Palmer.  Grostic’s apparently superhuman practice schedule  Case reports  Case anecdotes  Unsupported extrapolations from basic anatomy with oversimplified analogies for lay people | Subluxation  Innate Intelligence  Cephalocentroscope  Non-standard radiographic views |
| Kale (Michael U Kale) | Vitalism | ‘All other Upper Cervical Knee Chest programs on the market are direct derivatives of the Kale Program.”  ‘This information cannot be found anywhere else in any other program.’  ‘We are the Oldest and Most Preserved Knee Chest Organization in the World.’ | Spinal subluxation is primary cause of disease  Upper cervical spine most important  Radiographs must be obtained  The same set of radiographs is obtained on every patient  The radiographs are analysed the same way every time  Post treatment radiographs to ‘prove’ correction | X-ray machine  Radiographs | Silent killer subluxation  Case anecdotes  Patient testimonials  Practitioner testimonials    Unsupported extrapolations from basic anatomy with oversimplified analogies for lay people | The TIC institute  Non-standard radiographic views  ‘If the apparent listing does not clear the scan, then the listing will be re-evaluated and changed until the end result is a clear pattern on the scan.’ |
| Knee chest upper cervical specific (Robert Kessinger) | Vitalism | Unique categorisation of upper cervical subluxations (implication that they have more sophisticated understanding of subluxation)  6 week x-ray ‘bootcamp’ | Spinal subluxation is primary cause of disease  Upper cervical spine most important  Radiographs must be obtained  The same set of radiographs is obtained on every patient  The radiographs are analysed the same way every time | X-ray machine  Radiographs | 4 case reports (one in a PubMed indexed journal)  Case anecdotes and reference to BJs recordkeeping from 1935-1954 indicating 80-90% improvement or ‘full recovery’ from symptoms.  Medullary Lock concept (phrase coined by Mike Anderson)  Upper Cervical application of the HPA axis, HPT axis, Sympathetic Dominance | Subluxation  Non-standard radiographic views  Spizzerinctum |
| Logan Basic (Hugh Benedict Logan) | Vitalism | ‘…the secret of health and longevity has been solved… in Logan Basic Technique more than by all other asserted advances in healing methods since the beginning of time…’ | Spinal subluxation is primary cause of disease  Sacrum is most important  Radiographs must be obtained  The same set of radiographs is obtained on every patient  The radiographs are analysed the same way every time | X-ray machine  Radiographs | Case anecdotes  ‘as the sacrum goes, so goes the spine’ | Subluxation  X-ray marking system |
| Mears (Donald B Mears) | Vitalism | ‘…more reliable points of reference to determine cervical distortion more accurately. This in turn led to the need to modify the adjusting procedure used in the H.I.O. technique.’ | Spinal subluxation is primary cause of disease  Upper cervical spine most important  Radiographs must be obtained  The same set of radiographs is obtained on every patient  The radiographs are analysed the same way every time | X-ray machine  Radiographs |  | Subluxation |
| National Upper Cervical Chiropractic Association (NUCCA) (Ralph Gregory) | Vitalism | ‘…more biomechanical data than any other chiropractic entity concerning the atlas subluxation…’  ‘…measurable proof of the benefits of the chiropractic adjustment on the human body.’  ‘…accurately measuring and correcting upper cervical misalignments (subluxations) for more than sixty years.’ | Spinal subluxation is primary cause of disease  Upper cervical spine most important  Radiographs must be obtained  The same set of radiographs is obtained on every patient  The radiographs are analysed the same way every time  Post treatment radiographs to ‘prove’ correction | X-ray machine  Anatometer  Cephalometer | ‘Blood pressure study’ featured on GMA, PBS (on website) *Journal of Human Hypertension (*Atlas vertebra realignment and achievement of arterial pressure goal in hypertensive patients: a pilot study. Vol. 21, No. 5, 2007)  Case anecdotes  Unsupported extrapolations from basic anatomy | Non-standard radiographic views  Atlas Subluxation Complex Syndrome (ASC) or the ASC Syndrome  Anatometer  Cephalometer |
| Orthospinology (Society of Orthospinology) | Vitalism  Supernatural calling? | ‘…greatest health care procedure in the world.’  ‘…has helped millions of people overcome countless health problems.’ | Spinal subluxation is primary cause of disease  Upper cervical spine most important  Radiographs must be obtained  The same set of radiographs is obtained on every patient  The radiographs are analysed the same way every time  Post treatment radiographs to ‘prove’ correction | X-ray machine  Radiographs | Case reports  Patient testimonials  Case anecdotes  Unsupported extrapolations from basic anatomy with oversimplified analogies for lay people | Subluxation  Non-standard radiographic views |
| Palmer Upper Cervical Specific (HIO – Hole in One, Toggle Recoil) | Vitalism  Supernatural calling | BJ embraced the role of oracle to the profession and even compared himself to Christ.  ‘Knowing that our physical health and the intellectual progress of Innate depend upon the proper alignment of the skeletal frame, we feel it our bounded duty to replace any displaced bones so that physical and spiritual health, happiness, and the full fruition of earthly life may be fully enjoyed.’ | Spinal subluxation is primary cause of disease  Upper cervical spine most important  Radiographs must be obtained  The same set of radiographs is obtained on every patient  The radiographs are analysed the same way every time  Post treatment radiographs to ‘prove’ correction | X-ray machine  Radiographs | Case anecdotes  Patient testimonials  Practitioner testimonials    Unsupported extrapolations from basic anatomy with oversimplified analogies for lay people | Subluxation  Innate Intelligence  Spizzerinctum |
| Pettibon (Burl Pettibon)  Sharon Freese-Pettibon has been President of Pettibon System, Inc. since 1993 | Vitalism | Our goal is to enable you and your staff to reach new levels of success in delivering patient care while simultaneously maximizing the efficiency and profitability of your clinic.  Dozens of doctors, on the verge of leaving the profession, have reclaimed their calling because of the understanding and protocols which is now known as The Pettibon System.  ‘Conventional chiropractic x-ray procedures don't consider spinal soft tissue injuries. The Pettibon System's x-ray procedures do.’ | 3 phase care approach (Acute (14-21 days), with initial x-rays then again at the end of this phase, Rehabilitation and Correction (3 visits per week for at least 90 days), Maintenance and Supportive Care (1 visit per week for at least a year)  Training on home care equipment and procedures  Spinal subluxation is primary cause of disease  Upper cervical spine most important  Radiographs must be obtained  The same set of radiographs is obtained on every patient  The radiographs are analysed the same way every time  Post treatment radiographs to ‘prove’ correction | X-ray machine  Radiographs | Patient testimonials  Practitioner testimonials  Case anecdotes  Unsupported extrapolations from basic anatomy with oversimplified analogies for lay people  Misleading information about the dangers of ionizing radiation | Subluxation  Innate Intelligence  Non-standard radiographic views |
| Pierce System Results | Vitalism | ‘not a chiropractic technique like any others, but a systematic approach to correcting subluxations using the most advanced tools for spinal analysis and adjusting.’  ‘We don’t simply claim to provide the best chiropractic has to offer… we can back it up!’ | Spinal subluxation is primary cause of disease  Upper cervical spine most important  Radiographs must be obtained  The same set of radiographs is obtained on every patient  The radiographs are analysed the same way every time  Post treatment radiographs to ‘prove’ correction | X-ray machine  Radiographs | Case anecdotes | Subluxation  Atlas ‘out of compensation’  ‘Spinal visualizer’ believed to mean cineradiography.  ‘Master Level’ certification, not through a university |
| Spinal Orthopedic Neurological Advancement and Research (SONAR) (Thomas R Elliott, Jr) | Vitalism  Supernatural calling | ‘…far superior to other upper cervical techniques.’  ‘…we have patients who come in from around the country to receive upper cervical chiropractic care.’ | Spinal subluxation is primary cause of disease  Upper cervical spine most important  Radiographs must be obtained  The same set of radiographs is obtained on every patient  The radiographs are analysed the same way every time  Post treatment radiographs to ‘prove’ correction | X-ray machine  Radiographs | Case anecdotes  Unsupported extrapolations from basic anatomy | Subluxation |
| Spinal Stressology (Lowell Ward) | Vitalism  Supernatural calling | ‘I'm kind of a revolutionary scientist…’  ‘…we have documented more chiropractic cases than any before.’  more effective, longer-lasting adjustments  ChiroMan! (animated superhero) | Spinal subluxation is primary cause of disease  Radiographs must be obtained  The same set of radiographs is obtained on every patient  The radiographs are analysed the same way every time  Post treatment radiographs to ‘prove’ correction | X-ray machine  Radiographs | Patient testimonials  Case anecdotes  Unsupported extrapolations from basic anatomy with oversimplified analogies for lay people |  |
| Sutter Specific Atlas Correction (Max Sutter) | Vitalism  Supernatural calling | ‘The exact variation of the atlas subluxation in relation to its surrounding structures peculiar to the individual case must be accurately determined. This can only be done through a specialized X-ray technique and analysis.’  ‘Specific Atlas Correction deals with the specific location and correction of the cause of such interference within the nervous mechanism. It represents the latest development of scientific exactitude in the location and correction of the cause of disease within the body.’ | Spinal subluxation is primary cause of disease  Upper cervical spine most important  Radiographs must be obtained  The same set of radiographs is obtained on every patient  The radiographs are analysed the same way every time  Post treatment radiographs to ‘prove’ correction | X-ray machine  Radiographs | Case anecdotes  Unsupported extrapolations from basic anatomy | Subluxation  Innate intelligence  Non-standard radiographic views |
| Zimmerman (Arden D Zimmerman) | Vitalism | ‘…opens up a field for practice with absolutely no competition.’  ‘Due to the precision of the x-rays and other methods, ‘[the chiropractor] is trained to place the tip of the machine so accurately as to do that which has never been done before.’ | Spinal subluxation is primary cause of disease  Upper cervical spine most important  Radiographs must be obtained  The same set of radiographs is obtained on every patient  The radiographs are analysed the same way every time | X-ray machine  Radiographs | Patient testimonials  Practitioner testimonials  Unsupported extrapolations from basic anatomy with oversimplified analogies for lay people | Subluxation  Non-standard radiographic views |
